# Binding without Docking: Small-Molecule Binding Paths on Disordered Proteins

**DOI:** 10.1021/jacs.6c05891

**Published:** 2026-09-29

**Authors:** Adelie Louet, Gerhard Hummer, Michele Vendruscolo

**Affiliations:** † Department of Theoretical Biophysics, 28273 Max Planck Institute of Biophysics, Frankfurt am Main, 60438 Germany; ‡ Institute of Biophysics, Goethe University Frankfurt, Frankfurt am Main 60438, Germany; § Centre for Misfolding Diseases, Yusuf Hamied Department of Chemistry, 2152 University of Cambridge, Cambridge CB2 1EW, U.K.

## Abstract

Disordered proteins are challenging targets for drug design because they lack well-defined binding pockets. Small molecules can bind them, but when the bound state remains conformationally heterogeneous, these interactions are not readily characterized in terms of conventional binding pockets. Here, we use the concept of binding paths to describe how small molecules interact with disordered proteins. A binding path is a stochastic sequence of transient residue clusters that a ligand engages as it diffuses across a disordered protein, with each cluster forming and breaking up on time scales comparable to the overall residence time. We formalize this approach using a Markov state model (MSM), in which each state corresponds to a set of residues simultaneously contacting the ligand and the transition probabilities capture how the ligand moves between these sets. We apply this framework to the interactions of 10074-G5 with Aβ42 and of fasudil with α-synuclein, two disordered proteins associated with Alzheimer’s disease and Parkinson’s disease, respectively. The MSM enables both a dynamic mapping of transient binding sites and a calculation of dissociation constants consistent with experimental measurements. By generalizing the concept of static binding pockets to dynamic binding paths, this approach provides a quantitative framework for characterizing small-molecule binding by disordered proteins and for identifying druggable regions on these challenging targets.

## Graphical abstract

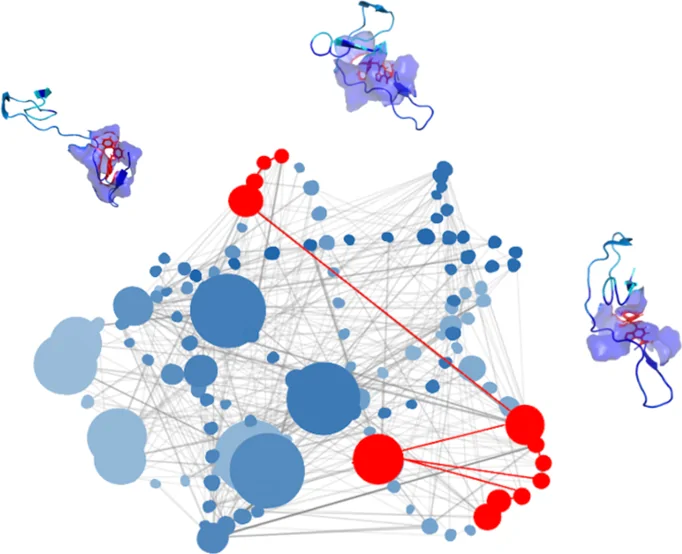

## Introduction

Disordered proteins make up about one-third of the human proteome and play essential roles in signaling, regulation, and molecular recognition.^1–4^ Although their dysregulation is central to a wide range of human diseases, they are still largely considered undruggable, a label that reflects our current lack of concepts and methods to discover effective drugs targeting them.^5–16^

Over the past two decades, a wide range of computational structure-based drug discovery pipelines, including virtual screening, molecular docking, molecular dynamics (MD) refinement and free-energy perturbation, have flourished because many disease-relevant proteins present well-defined binding pockets.^17–22^ This strategy has generated approved drugs, from kinase inhibitors in oncology to the pharmacological chaperone tafamidis, which fits into a thyroid-hormone-like cleft in transthyretin and prevents its misfolding.^23,24^

In the case of disordered proteins, although stable binding pockets do not typically exist,^5–15^ it has been recently shown that the binding of small molecules can arise through the formation of ordered tertiary contacts upon binding.^25,26^ Insight into a possible molecular mechanism of binding comes from previous molecular dynamics (MD) studies of folded protein–ligand systems, in which ligand recognition has been reconstructed as a sequence of metastable intermediates and diffusion events rather than direct insertion into a binding pocket.^27–32^

An analogous picture has emerged for disordered proteins, where, using MD simulations validated by nuclear magnetic resonance (NMR) spectroscopy, small molecules targeting the disordered N-terminal transactivation domain of the androgen receptor were identified and shown to induce the formation of compact, α-helical, molten-globule-like conformations.^25,26^

In cases where the bound state remains disordered, NMR relaxation dispersion and small-angle X-ray scattering showed that ligand binding to the disordered D2 domain of p27Kip1 shifts the population among pre-existing low-energy conformations already present in the unbound ensemble,^33^ rather than expanding the disorder of the chain.^6^ Long-time-scale MD simulations and NMR studies of fasudil binding to α-synuclein revealed a dynamic shuttling mechanism, in which the ligand engages the disordered C-terminal region through a network of weak, transient charge–charge and π-stacking contacts rather than adopting a single fixed pose.^34^ This behavior was consistent with residue-specific NMR chemical shift perturbations and validated by the design of fasudil analogues spanning a range of binding affinities.^34^ Using ^19^F NMR spin relaxation, 5-fluoroindole was shown to bind disordered domains of the hepatitis C virus protein NS5A with μM affinity while remaining highly mobile in the bound state, with a rotational correlation time only marginally longer than in its free form.^35^ This confirms a bound state of persistent, rapid fluctuations, consistent with the view that such interactions are governed by ensembles of transient, loosely defined contacts rather than stable pockets.^6,34–36^ Finally, the small-molecule inhibitor 10074-G5 (referred to as G5 in the following) binds the monomeric state of Aβ42 with low μM affinity and inhibits its aggregation, forming transient hydrophobic, charge–charge, aromatic π–π stacking and hydrogen-bonding contacts; complex formation is favored by an increase in the conformational entropy of Aβ42, which becomes more disordered in the bound state.^37,38^

These observations point to a common mechanistic picture. Rather than occupying a single, stable pocket, a small molecule diffuses across a disordered protein, engaging a series of transiently formed contact sites.^34–41^ Quantitative approaches that jointly enumerate these transient sites, quantify the rates of interconversion between them, and relate them to the overall binding affinity remain challenging.

Here, we address this gap by casting the disordered binding mechanism as a Markov state model (MSM).^29,30,40–45^ In our specific implementation, each state in the MSM corresponds to a cluster of residues simultaneously in contact with the ligand, and the transition probabilities encode how the ligand moves between these clusters over time. We construct the MSM by integrating MD simulations with stochastic analysis of residue-level contacts and network-based community detection, and we apply it to two systems for which the disordered binding mechanism has been experimentally characterized: G5 binding to Aβ42 and fasudil binding to α-synuclein. From the MSM, we extract binding paths, as the stochastic routes that a small molecule traces across a disordered protein as it sequentially engages overlapping residue clusters. These binding paths provide a dynamic generalization of static binding pockets, enabling the identification of targetable regions that are not readily visible to conventional pocket-based descriptions. We use the term binding path to describe the trajectory of a ligand as it diffuses across a disordered protein surface, represented here by the time-ordered sequence of residue-contact states visited in the MSM. The Markov property applies to this coarse-grained representation, which we introduce to obtain a tractable kinetic description. It is not meant to imply that the underlying continuous diffusion process itself is memoryless. In graph-theoretical terms, the trajectory corresponds to a random walk on the contact-state network, but our focus is on its biophysical interpretation as a ligand-binding process.

Our results indicate that the conceptual shift from static binding pockets to dynamic binding paths offers an understanding of how small molecules interact with disordered proteins through a disordered binding mechanism. The approach requires sufficient sampling to resolve metastable contact states and their interconversion, but it does not require a predefined binding pocket or a single dominant-bound conformation. We therefore report convergence diagnostics, including the Chapman–Kolmogorov test, to assess when a binding path characterization is reliable.

## Results

### Discrete Clustering Approaches Reveal the Absence of Separable Binding States in Aβ42

To establish whether conventional state-based clustering could resolve discrete binding pockets in the interaction between Aβ42 and G5, we applied VAMPNet, a deep learning framework that extracts slow dynamical modes from time-lagged MD data and provides a principled route to metastable state definition for MSM construction.^46^

We provided as input the time series of distances between the center of mass of G5 and the Cα atoms of Aβ42 and employed a two-level hierarchical clustering procedure, with each level producing four clusters and yielding 16 discrete states in total. The resulting state assignment was highly imbalanced, as more than 90% of frames were mapped to a single state (state 2) (Figures 1 and S1). Smaller clusters captured only marginal fractions of the trajectory, with no evidence of kinetically separable bound configurations of comparable population.

**Figure 1:**
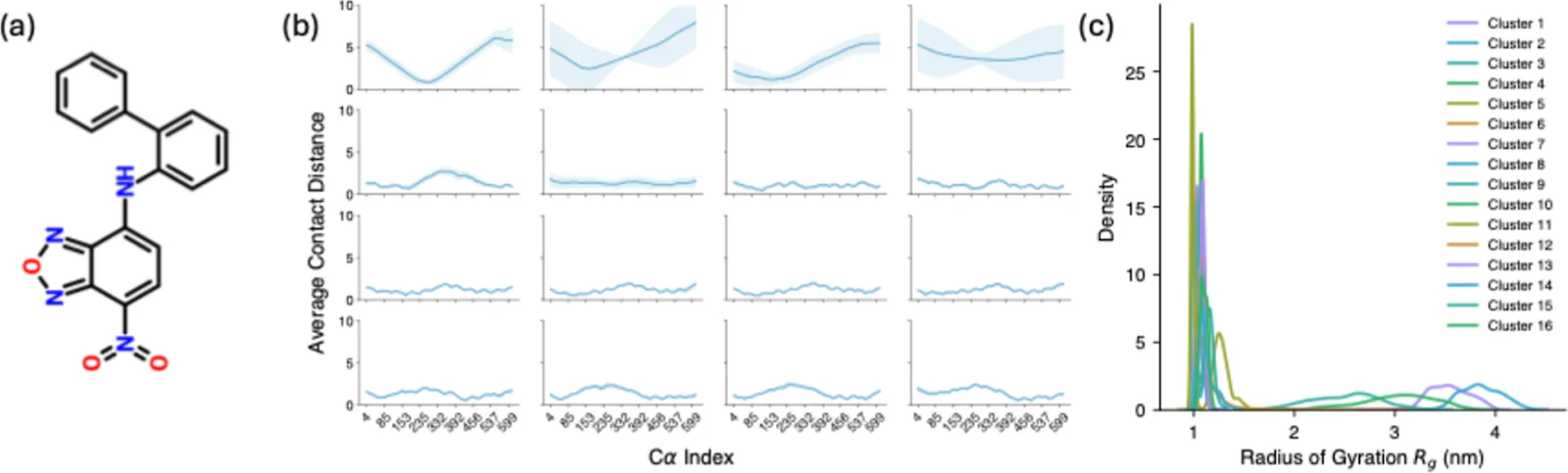
Discrete clustering approaches reveal the absence of separable binding states in Aβ42. (a) Chemical structure of the small molecule 10074-G5 (biphenyl-2-yl-(7-nitro-benzo­[1,2,5]­oxadiazol-4-yl)-amine; molecular formula C_18_H_12_N_4_O_3_), referred to as G5 throughout. (b) Mean ligand-Cα distance profiles (shaded bands: ± one standard deviation) for each of the 16 microstates obtained from hierarchical VAMPNet clustering. The few low-population clusters show structured profiles with residue-specific distance patterns, while the dominant cluster (cluster 2, containing >90% of frames) shows a featureless profile with large variance, indicating no preferred binding geometry. (c) Distributions of the radius of gyration of Aβ42 across clusters. The dominant cluster spans a broad range of protein compactness, further confirming that it does not correspond to a structurally defined binding state.

This result may reflect a fundamental property of the system, where the interaction between G5 and Aβ42 does not organize itself into a small number of discrete, well-separated metastable states. Instead, the ligand samples a broad, continuously varying set of interaction geometries as it diffuses across the disordered protein, without settling into stable, recurrent binding pockets of the kind that would produce balanced cluster populations. The dominance of a single cluster therefore reveals that the binding landscape of this disordered complex is intrinsically diffuse, and that frameworks premised on the existence of discrete pockets will not readily describe it.

This observation motivates a fundamental shift in the modeling strategy. We ask how the stochastic diffusion of the ligand across the protein can be described directly. In the sections that follow, we develop a representation based on residue-level contact transitions (the handoff matrix) and build from it an MSM whose states are defined not as global binding configurations but as local residue clusters transiently engaged by the ligand (Figure S2). This approach accommodates the continuous and probabilistic nature of binding to disordered proteins without assuming the existence of stable pockets.

### Stochastic Nature of the Binding between G5 and Aβ42

To better understand the challenges in the clustering binding of pockets in a disordered protein, we analyzed how G5 diffuses along Aβ42 by forming transient contacts with different side chains. This behavior was characterized by generating a time series that tracks whether each individual residue is in contact with G5 at every frame of an MD trajectory (Figure 2). The resulting time series revealed a stochastic pattern of interactions, consistent with previous reports that G5 does not preferentially bind to a specific, well-defined site, such as a stable cavity or cleft, typically associated with structured proteins.^37,38^ Instead, the ligand dynamically diffuses across Aβ42, displaying generalized affinity for multiple residues rather than settling into a single, persistent binding pocket. This behavior highlights the limitations of traditional clustering approaches, which assume discrete, metastable binding states, and underscores the need for models that can account for the continuous and probabilistic nature of binding to disordered proteins.

**Figure 2:**
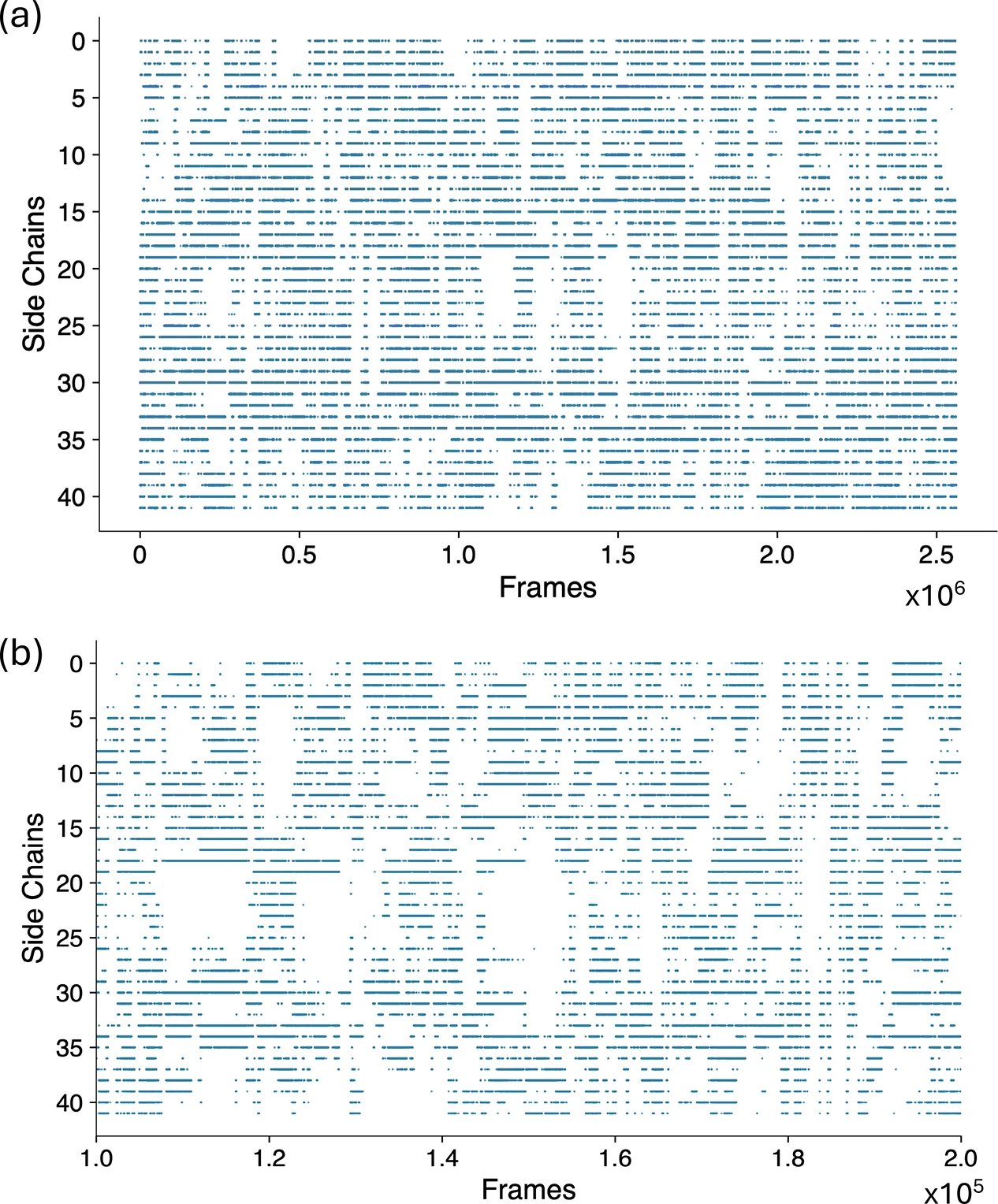
Stochastic contact patterns between G5 and individual Aβ42 residues. (a) Contact map across the full trajectory (2.8 × 10^6^ frames). Each dot indicates that the ligand is within 3 Å of at least one atom of the indicated residue at that frame. Contacts are distributed across the entire sequence but are most persistent near residues 3–8 and 28–40. (b) Expanded view of frames 1,000,000–2,000,000, showing that multiple residues are engaged simultaneously at any given time and that the set of contacted residues changes continuously, consistent with a diffusion mechanism rather than binding to discrete, recurrent pockets.

### Residue-Level Mapping of the Stochastic Binding Using a Handoff Matrix

From the analysis described above, two key observations emerged. First, the inability to identify discrete clusters appears to arise from the vast number of states in which disordered proteins can exist. Second, a dynamic shuttling mechanism suggests that ligands diffuse rapidly on the protein without populating stable binding pockets. Our approach involved exploring three-dimensional and highly dynamic conformations, focusing on the continuous interaction over the trajectory rather than isolated snapshots at individual time points.

To better understand how a small-molecule ligand dynamically interacts with a disordered protein, we developed a representation that captures how the ligand transitions between residue contacts over time. We refer to this representation as the handoff matrix, which encodes the frequency with which the ligand moves from one residue to another during the MD simulations. Specifically, for Aβ42, the handoff matrix is a 42 × 42 matrix H, where each element H_ij_ represents the number of observed transitions in which the ligand is in contact with residue *i* at one time step and with residue *j* at the subsequent time step (Figure 3). This representation enabled us to capture the inherently stochastic behavior of G5 as it diffuses across Aβ42, forming and breaking transient contacts with different residues. Importantly, this representation shifts the perspective from discrete, static binding events to a dynamic process in which interactions are continuously redistributed across the protein.

**Figure 3:**
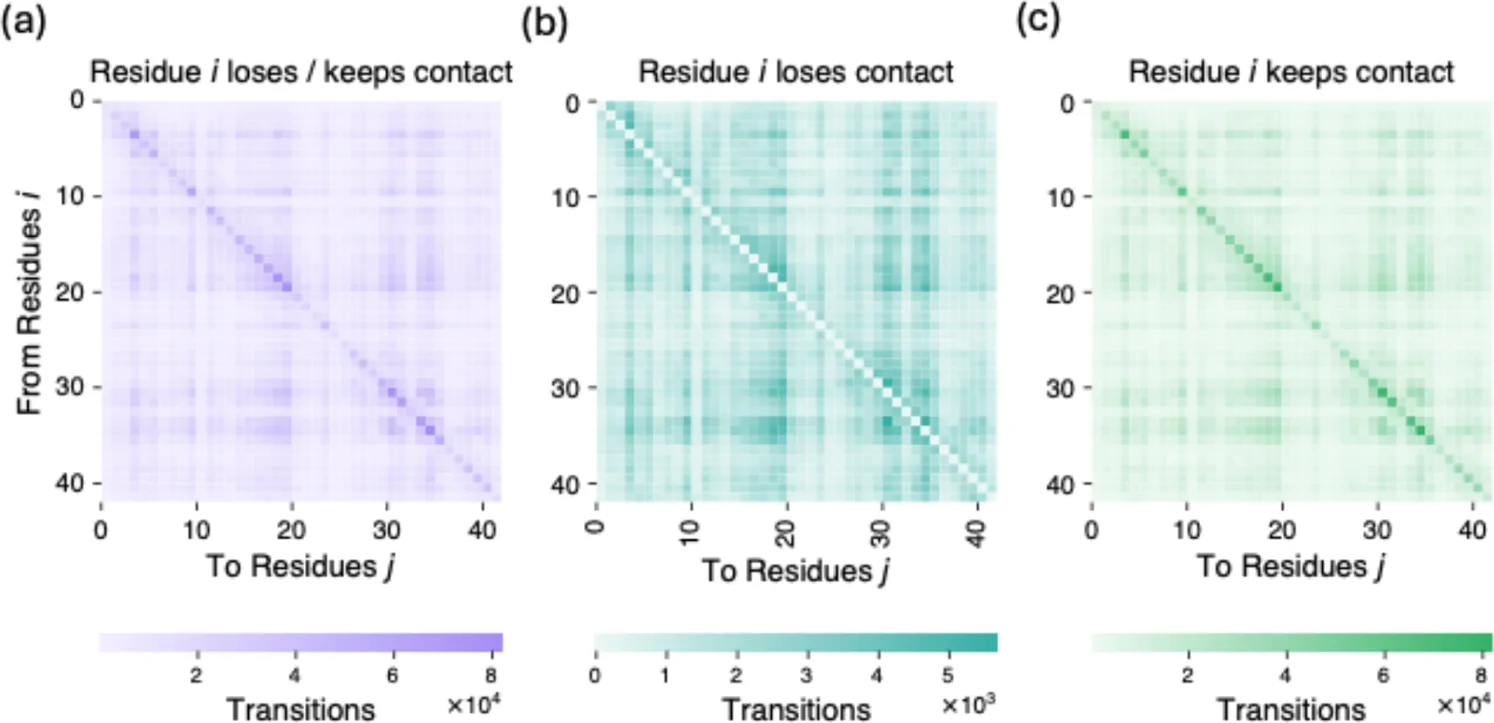
Handoff matrices quantifying residue-to-residue ligand transfer on Aβ42. Each matrix element H_ij_ records the number of transitions (colorbar) in which G5 contacts residue *i* at frame *t* and residue *j* at frame *t+*1, decomposed into three cases. (a) All transitions, regardless of whether residue i retains contact (case A). (b) Strict handoffs, in which residue *i* loses contact as residue *j* gains it (case B). (c) Additive contacts, in which residue *i* remains bound while residue *j* is newly engaged (case C). Case A is the sum of cases B and C. The strong diagonal in panels (a) and (c) indicates that contacts persist over consecutive frames, while the off-diagonal hotspots (concentrated around residues 3–8, 28–35, and 36–41) identify the regions between which the ligand most frequently transfers. The sparser signal in panel (b) compared to that in (c) indicates that additive contact accumulation is more common than clean residue-to-residue handoff, consistent with the gliding mechanism characterized in Figure 4.

To characterize the nature of these transitions, we analyzed the hand-off events in terms of three specific cases. In case A, we recorded all transitions in which residue *i* was in contact with the ligand at time *t*, and residue *j* was in contact at time *t+*1, regardless of whether residue *i* retained its contact (Figure 3a). Case B restricted this calculation to events in which residue *i* lost its contact while residue *j* gained a contact, representing a strict handoff (Figure 3b). Case C captured scenarios in which residue *i* maintained its contact while residue *j* was newly engaged, highlighting additive interactions rather than replacements (Figure 3c).

The resulting handoff matrices reveal key binding hotspots on Aβ42. These are regions where the ligand frequently transitions between specific pairs of residues. These hotspots reflect areas of elevated interaction probability and serve as a dynamic analogue to classical binding pockets. By averaging over matrix elements corresponding to specific groups of residues, we can define effective transition probabilities that are then incorporated into the MSM framework. This approach thus provides a mechanistic basis for identifying targetable regions on disordered proteins and for describing the binding behavior of a ligand in terms of continuous diffusion rather than discrete docking.

### Definition of a Markov State Model of the Stochastic Binding

To capture the dynamics of ligand binding to Aβ42, we constructed an MSM based on the transitions between residue clusters that interact with the ligand over time. In contrast to traditional approaches that define states based on global protein conformations or static binding pockets, we defined states as n-plets, which are sets of residues simultaneously in contact with the ligand at a given time point. We systematically enumerated all residue combinations of sizes two to five (i.e., doublets to quintuplets) that formed simultaneous contacts with the ligand throughout the simulation. Each unique combination was treated as a distinct MSM state. As the simulation progressed, we tracked how the ligand transitioned between these states by recording which sets of residues lost contact and which new sets gained contact at each time step (Figure 4a).

**Figure 4:**
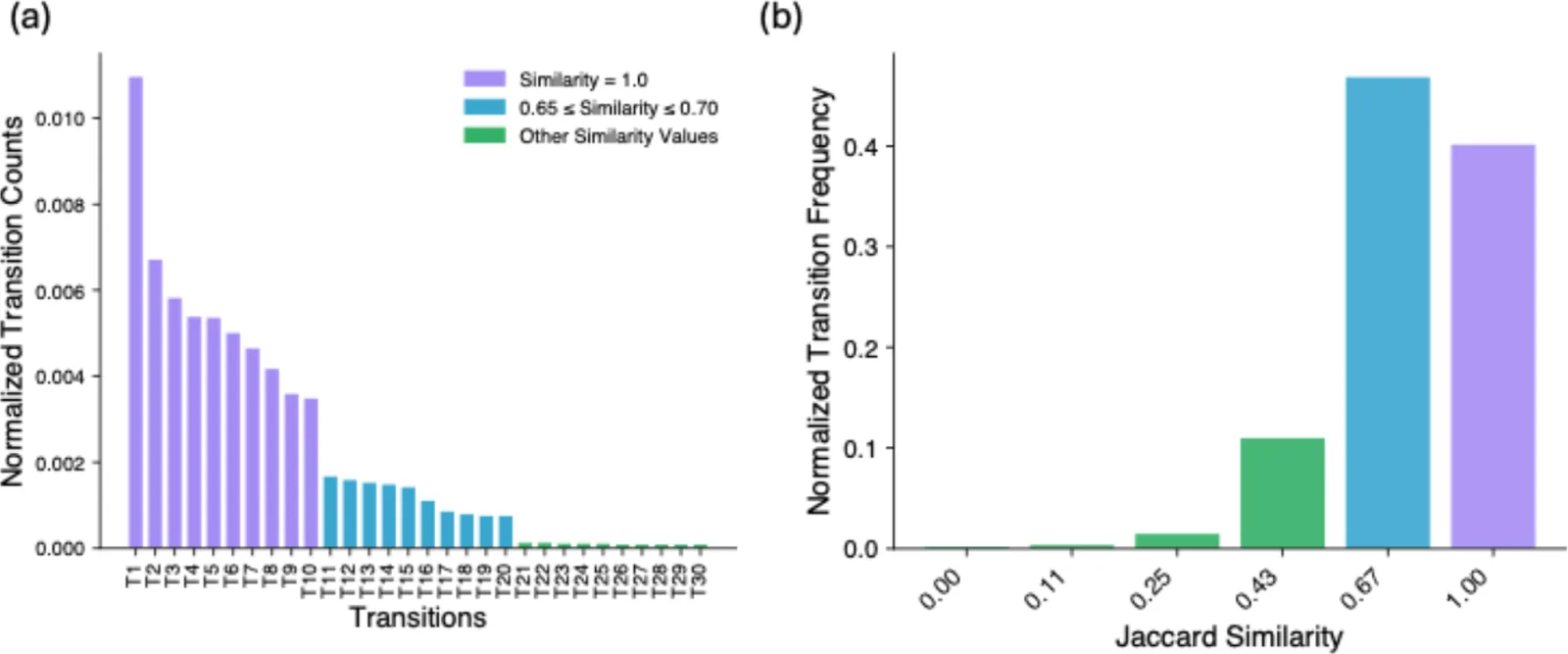
Transition frequency analysis reveals a gliding mechanism with high-contact persistence. (a) The 30 most frequent individual transitions, colored by similarity class. T1–T10 (purple) are self-transitions; T11 onward involves transitions between distinct but highly overlapping quintuplets (see Table S1 for residue identities). (b) Distribution of normalized transition frequencies between consecutive quintuplet states, binned by Jaccard similarity. For quintuplets, similarity values are discrete: 1.0 (identical state, i.e., self-transition), 0.67 (four shared residues), 0.43 (three shared), 0.25 (two shared), 0.11 (one shared), and 0.0 (no overlap). Self-transitions (Jaccard = 1.0) account for approximately 40% of all transitions, indicating high-contact persistence. Among transitions between distinct states, single-residue exchanges (Jaccard = 0.67) strongly dominate over long-range hops (Jaccard ≤0.43, collectively ∼7%), supporting a gliding mechanism.

This approach allowed us to construct a transition matrix, where each element represents the frequency of transitions between specific n-plets. The model distinguishes between two types of transitions: gliding transitions, where the ligand moves between similar clusters (e.g., with high residue overlap), and hopping transitions, where the ligand jumps between dissimilar regions on the protein. To quantify this distinction, we computed the Jaccard similarity between consecutive n-plets. We found that most transitions occurred between highly similar residue sets (Jaccard similarity ≥0.66), supporting a gliding mechanism of ligand diffusion across the protein (Figure 4b). In contrast, transitions between dissimilar n-plets were rare (approximately 7%), indicating that long-range hops are infrequent (Figure 4b). To confirm that this gliding-dominated behavior is a general feature of disordered protein–ligand binding rather than specific to Aβ42, we performed the same Jaccard-similarity and flux analysis across 45 IDP–ligand trajectories spanning 12 chemically and structurally diverse intrinsically disordered proteins and found that high-overlap gliding transitions consistently carry the majority of the flux in every system (Figure S3).

By modeling the stochastic movement of the ligand between overlapping contact clusters, this MSM framework provides a state-based representation of the binding process, well suited to the disordered and dynamic nature of Aβ42. The resulting model offers kinetic and thermodynamic insights into how a small molecule engages with the disordered protein.

### Master Equation Analysis of the Markov State Model

To quantitatively describe the binding dynamics captured by the MSM, we analyzed the system using a master equation formalism. This framework enabled us to compute how the probability distribution of ligand-bound states evolves over time, based on the transition rates between states. Each MSM state corresponds to an n-plet (e.g., a quintuplet) of residues simultaneously in contact with the ligand. The full set of these states defines the space in which the ligand diffuses. For Aβ42, when considering quintuplets, this procedure identified a total of 13,034 states. The transitions between these states, recorded from an MD trajectory, define the transition rate matrix used in the master equation, which describes the time evolution of the probabilities of the states of the system.^47^ The long-time behavior of the system converges to a stationary distribution π^eq^ = [π_1_^eq^, ...,π_n_^eq^], which corresponds to the equilibrium population of each state (see Supporting Information).

To validate the accuracy of the MSM, we performed a Chapman–Kolmogorov (CK) test,^42^ comparing the predicted evolution of state probabilities with the actual data from the simulation across multiple lag times. We found that the agreement between predicted and observed dynamics improved as the size of the residue clusters increased, with quintuplets providing the best CK agreement (Figure 5). These results suggest that larger n-particles yield MSMs with better Markovian consistency under the CK test.

**Figure 5:**
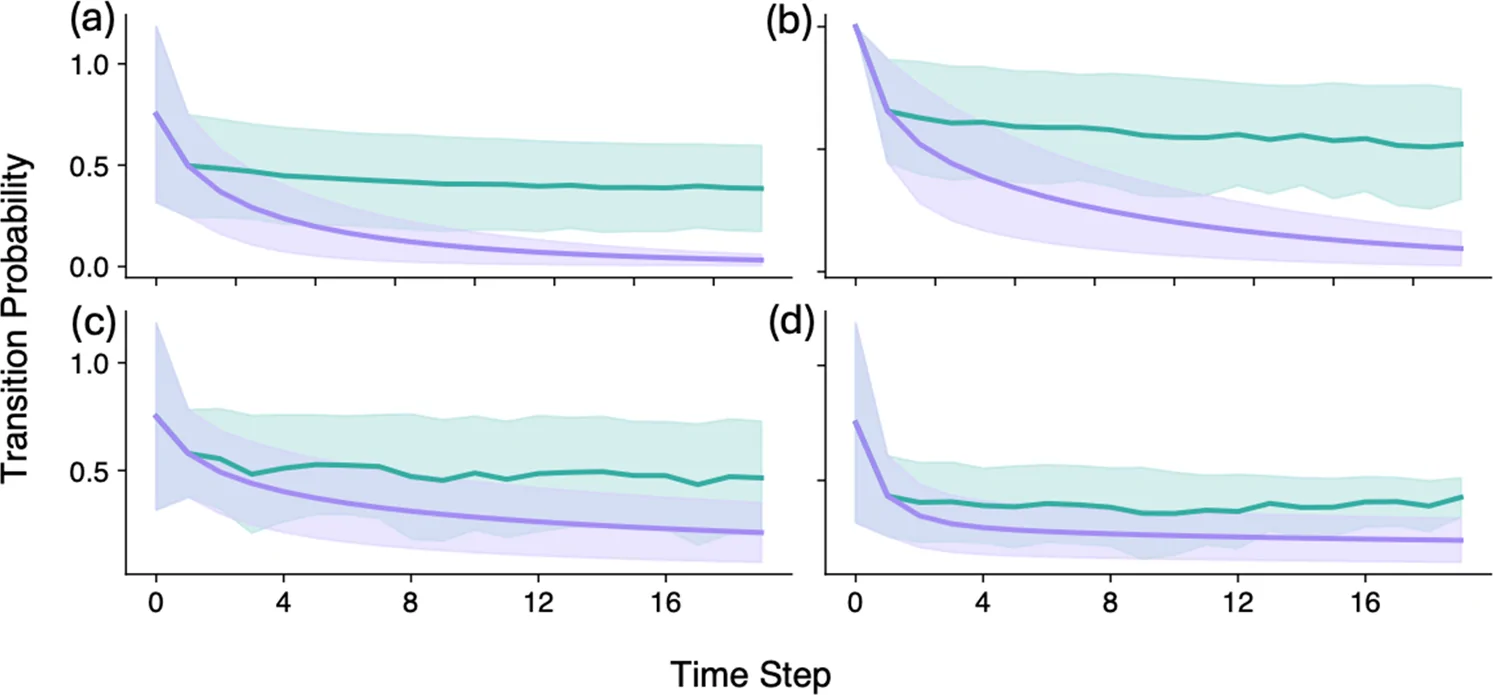
Chapman–Kolmogorov validation of the MSM for different n-plet sizes. (a–d) Each panel tests the self-consistency of the MSM by comparing state populations propagated by the transition matrix at lag time τ over multiple steps (purple, “Mean Predicted”) with populations estimated directly from the trajectory at the corresponding longer lag times (green, “Mean Empirical”); shaded bands show 95% confidence intervals. Panels show the test for doublets (a), triplets (b), quadruplets (c), and quintuplets (d). Agreement between predicted and empirical curves improves with increasing n-plet size, with quintuplets showing the closest match, supporting their use as the MSM state definition. However, systematic deviations persist at longer time steps even for quintuplets, and the wide confidence bands (particularly for smaller n-plets) reflect limited statistical power.

### Visualization of the MSM through Knowledge Graphs

To visualize an MSM, we constructed a knowledge graph, which is a type of network diagram that represents entities (nodes) and their relationships (edges). In our context, each node corresponds to a quintuplet of residues simultaneously in contact with the ligand, representing a unique state in the MSM (Figure 6a). Edges between nodes indicate observed transitions between these states based on an MD trajectory. The spatial arrangement of nodes in the graph reflects the frequency of transitions: nodes that are closer together correspond to states between which the ligand undergoes more frequent transitions. This visualization provides a high-level map of the binding landscape, revealing both the structural connectivity and the dynamic relationships among the ligand-bound states.

**Figure 6:**
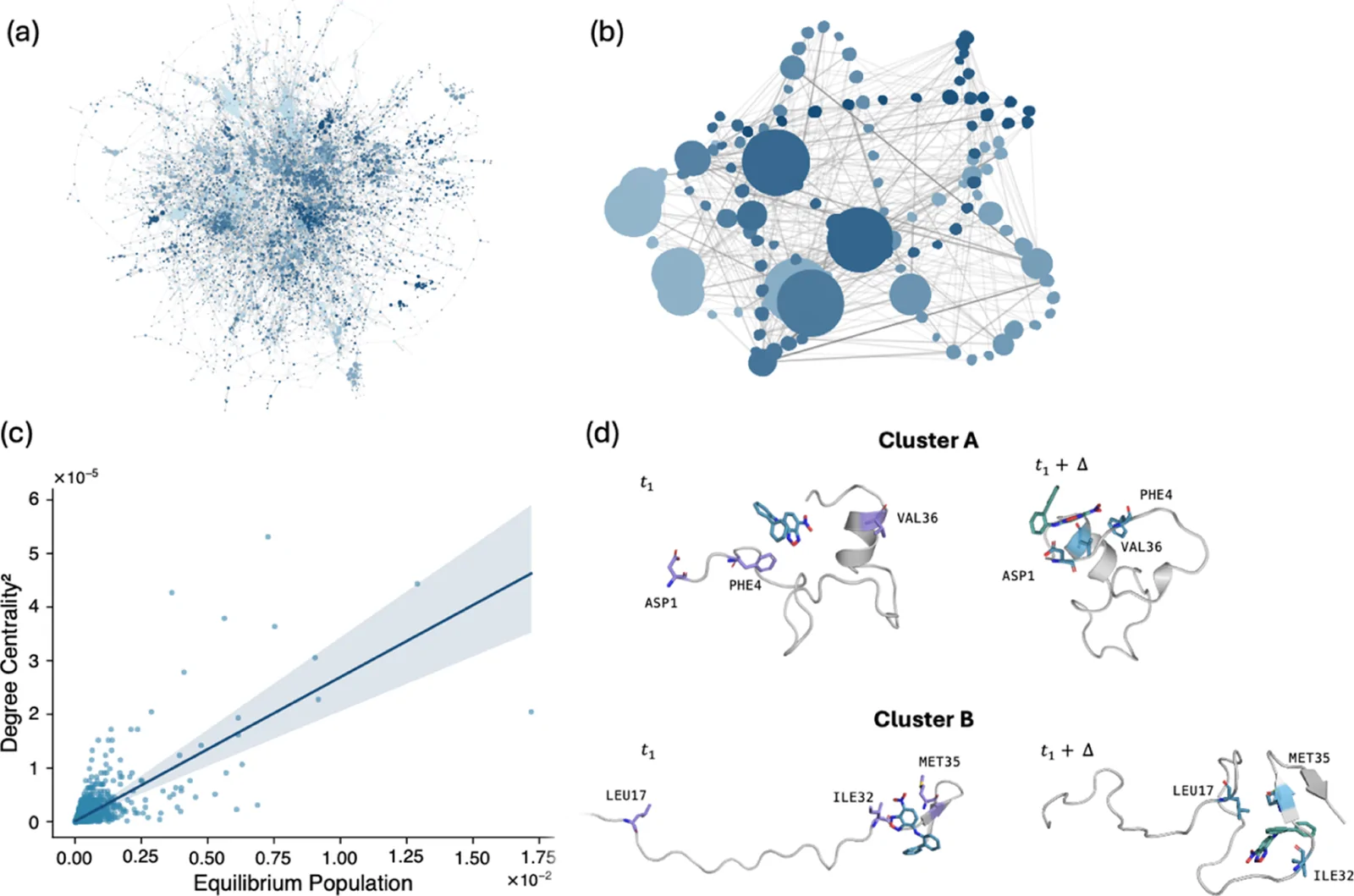
Network analysis of the MSM state space for Aβ42-G5. (a) Full microstate network: each of the 13,034 quintuplet states is a node, with edges representing observed transitions. Node size is proportional to degree centrality; colors denote Louvain communities (resolution = 3). The dense, interconnected topology reflects the diffuse binding landscape. (b) Macrostate network obtained by aggregating nodes within each community into a single representative node, reducing the network to 146 macrostates. Node size reflects the summed equilibrium population of the community, and edges represent intercommunity transition flux. (c) Scatter plot of degree centrality (*y*-axis) versus equilibrium population (*x*-axis) for individual quintuplet states, showing a positive correlation (trend line). Because K_*i*_^*e*^ is inversely proportional to equilibrium population (eq 1), this indicates that more highly connected states also tend to bind the ligand more strongly. (d) Representative snapshots from two macrostate clusters (cluster A and cluster B), each shown at two consecutive time points (t_1_ and t_2_ + Δ).

Quintuplets were then clustered using the Louvain algorithm, which is a greedy algorithm that iteratively groups nodes into communities by maximizing modularity and then builds a coarse-grained network of those communities to uncover hierarchical structure.^48^ Edges between nodes represent observed transitions between states, and the proximity of the nodes reflects the frequency of transitions between them. Nodes were grouped into communities, with each community representing a set of closely connected states (Figure 6b). In this way, the original 13,034 states were clustered into 146 macrostates. The size of each node is proportional to its degree centrality, reflecting how frequently that state participates in transitions within the network (Figure 6a).

This graph-based representation highlights both the dynamics of the state transitions and the affinity landscape of the system. Notably, degree centrality correlates with the state-specific affinity constant

1 $$K_{i}^{e}=\frac{p_{u}^{eq}}{p_{i}^{eq}}$$

where *p*_*u*_^eq^ and *p*_*i*_^eq^ are MD-derived probabilities of the unbound state and of state *i*, such that more highly connected states tend to exhibit stronger ligand binding (Figure 6c). States with higher equilibrium populations correspond to lower *K*_*i*_^e^ values. Quintuplets were chosen as the representative unit for this analysis based on the outcome of the Chapman–Kolmogorov (CK) test, which indicated that five-residue clusters provided the most robust model.

Additionally, we assessed the intra- and intercluster radius of gyration (*R*_g_) and root-mean-square deviation (RMSD) to examine structural variability between and within clusters (Figure S4). Representative structural snapshots from two macrostate communities make this behavior explicit: within each community, the set of residues contacting G5 (light blue) is visibly reorganized between two consecutive time points, confirming that states grouped together by the MSM share a binding region but not a fixed contact pattern (Figure 6d).

To illustrate the concept of binding paths, we extracted a representative trajectory segment in which G5 traverses multiple macrostate communities on Aβ42 (Figure 7). The top row shows structural snapshots at three successive time points: the ligand occupies distinct regions in each, with the local binding region (purple) reshaping as the contacted residue cluster changes. The middle row maps these same time points onto the full microstate network, highlighting the visited states in red against the background topology; the bottom row shows the corresponding positions on the macrostate network, with intercommunity transitions drawn as red edges. The ligand does not remain within a single community but instead crosses between neighboring communities through the high-connectivity nodes identified in Figure 6c, consistent with the gliding mechanism characterized in Figure 4. This trajectory segment illustrates how a binding path emerges as a directed walk through the MSM state space, not a random exploration of the full network, but a sequence of transitions concentrated among kinetically linked, high-population communities.

**Figure 7:**
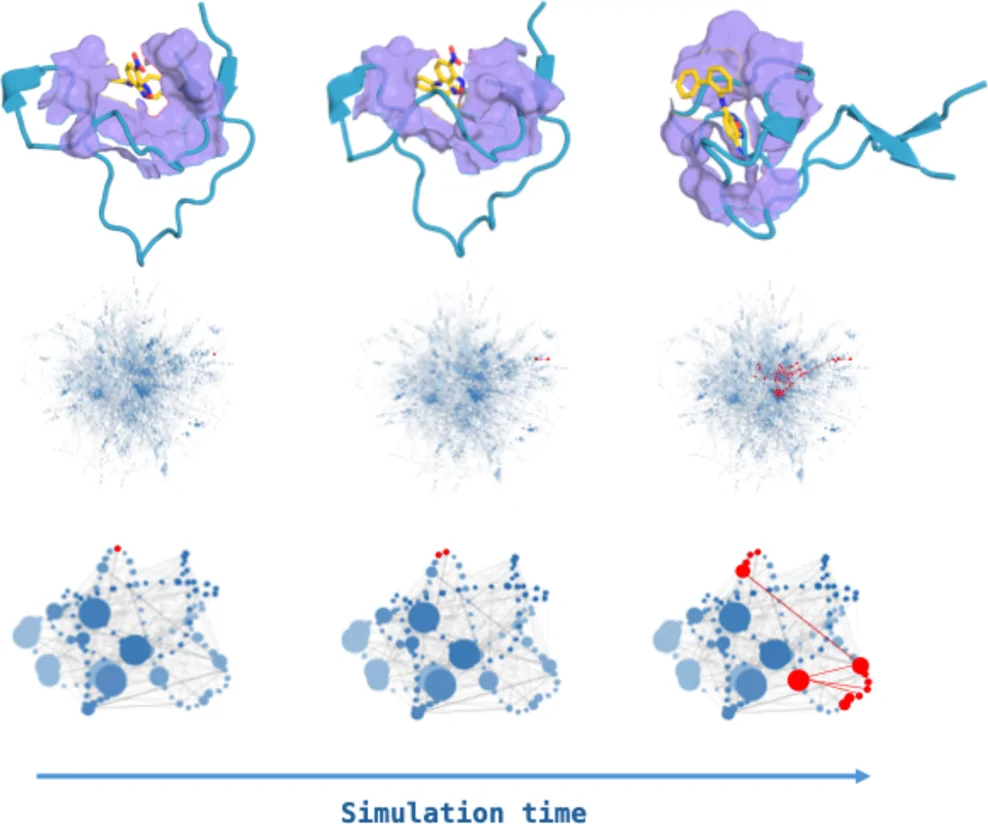
Visualization of a representative binding path for G5 on Aβ42. Three time points along the trajectory (left to right) are shown at three levels of representation. Top: structural snapshots showing the protein (blue ribbon) and ligand (yellow sticks) with the local binding region (purple). Middle: the full microstate network (Figure 6a), with the states visited at each time point highlighted in red. Bottom: the macrostate network (Figure 6b), with the currently occupied communities highlighted in red and the transitions between them shown as red edges. The ligand moves across different regions of Aβ42 over this interval, illustrating how a binding path traverses multiple communities in the network.

### Binding Affinity Calculations

We calculated the overall binding affinity using two complementary approaches. First, we estimated K_D_ from the MD simulations, through the total number of frames in which the ligand was in an unbound state versus any bound state (eq S2), yielding a K_D_ of 49 ± 4 μM for Aβ42-G5. The error was estimated by repeating the calculation 10 times with a sliding window covering half the trajectory at 10 different intervals. This value is broadly consistent with experimental measurements of 6 μM (by biolayer interferometry) and 40 μM (by ThT fluorescence) previously reported.^37,38^ Second, we derived the dissociation constant from the equilibrium distribution of the MSM, where an unbound state was included (eq S3), again yielding a K_D_ of 47 μM for Aβ42-G5.

To assess the robustness of our model across disordered proteins of different lengths, we applied our approach to a previously reported trajectory^34^ of the α-synuclein C-terminus in complex with small molecules. Using the published 60 μs atomistic MD simulation performed with the a99SB-disp force field,^49^ and following the same analysis protocol described above, we obtained equilibrium dissociation constants of 25 μM, 26 μM, and 18 μM for C-terminal α-synuclein bound to ligands 40, 50, and 12, respectively. To evaluate the dependence of these results on system size, we further applied the same analysis to full-length α-synuclein in complex with the small molecule fasudil, yielding an equilibrium dissociation constant of 60 μM (Figure 8).

**Figure 8:**
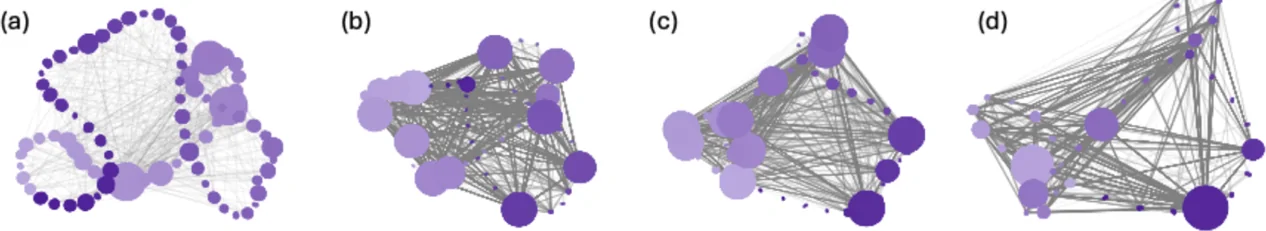
Macrostate networks for four α-synuclein-ligand systems. Each panel shows the Louvain-clustered macrostate network for a different ligand, constructed using the same protocol as Figure 6b. (a) Fasudil bound to full-length α-synuclein (140 residues; 68 macrostates; computed K_D_ = 60 μM). (b–d) Ligands 12, 50, and 40 bound to the C-terminal region of α-synuclein (38, 39, and 37 macrostates; computed K_D_ = 18, 26, and 25 μM, respectively). The node size is proportional to the equilibrium population. The denser and more interconnected network for ligand 12, which also has the lowest K_D_, is consistent with the correlation between network connectivity and binding affinity observed for Aβ42 (Figure 6c).

This analysis enables a quantitative ranking of binding states according to their stability and affinity (Table S1), providing a thermodynamic interpretation of the interaction of a ligand with the disordered protein. The high-affinity states identified by this ranking are characterized by elevated hydrophobicity, reduced solvent-accessible surface area at the contact set, and modest transient local ordering of the contacted residues, with no single feature dominant (Figure S5). At the level of individual residues, these high-affinity states localize to the central hydrophobic cluster (residues 17–21) and the C-terminal region (residues 30–41), and the ligand preferentially engages residues at moments when they adopt a transient α-helical or β-sheet structure, indicating that binding is associated with, and may help stabilize, transient local ordering of the disordered chain (Figure S6). Moreover, the MSM captures the collective behavior of multiple states, including self-transitions (where the ligand remains in the same state) and the possibility of the ligand being bound to multiple residue clusters simultaneously.

## Discussion

In this study, we investigated the complex and dynamic nature of small-molecule binding to disordered proteins. Our analysis addresses the challenges in applying traditional drug discovery approaches to disordered proteins that typically rely on identifying stable binding pockets. These methods are often inadequate for disordered proteins because of their inherent lack of persistent structural features.

Toward this goal, we developed an MSM framework that defines the binding mechanism in terms of a series of transient binding pockets interconnected through a network of transition pathways. Our results show that the formation of a disordered complex between a small molecule and a disordered protein can be effectively captured using this stochastic approach. In the cases of Aβ42 and α-synuclein, we identified regions on the protein where the ligands frequently interact with specific clusters of residues. Rather than binding to a fixed site, the ligands exhibit diffusive behavior, transitioning smoothly between related residue clusters.

By employing a stochastic modeling approach in terms of an MSM, we quantified both the binding affinities and transition frequencies between these dynamic states, providing a comprehensive view of the behavior of the ligands. Visualizing the MSM as a knowledge graph further revealed the intricate relationships among the binding states and illustrated the overall structure of the binding landscape. The integration of long-time-scale MD simulations, community detection algorithms, and Markov state modeling underscores the effectiveness of combining advanced computational techniques to study disordered proteins. This approach identifies dynamic binding hot spots and offers a quantitative framework for analyzing both the thermodynamic and kinetic aspects of ligand binding.

The concept of binding paths could help explain binding specificity upon further study. Rather than targeting a single, well-defined pocket, a ligand can be designed to follow the characteristic sequence of transient binding pockets that a particular disordered protein presents as it moves. Designing a small molecule whose chemical groups engage those clusters in just the right sequence could, in principle, contribute to selectivity if the path features are sufficiently distinct across the proteins. Moreover, the specificity conferred by binding paths is derived not only from thermodynamic complementarity but also from kinetic matching. A ligand whose on- and off-rates are tuned to the measured transition frequencies between hotspots becomes effectively bound on its intended path. It also samples other proteins that are too inefficient to bind appreciably. Finally, once the high-probability binding paths of a target are computationally mapped, one can extract a fingerprint of the residue-transition patterns. This fingerprint then guides fragment-based growth or focused library screening toward chemotypes uniquely matched to the path. In this way, the concept of dynamic binding paths suggests a design hypothesis in which dynamic contact patterns could be exploited to bias recognition toward target disordered proteins.

To provide more context to our findings, we note the analogy between our results and those reported in a recent article that presented a t-SNE-based clustering framework for analyzing the conformational ensembles of disordered proteins and their interactions with small molecules.^36^ A key conclusion of that study was that, despite the structural heterogeneity of disordered proteins, ligand binding can lead to the emergence of metastable substates that are structurally and energetically distinct. In the case of Aβ42, they found that G5 bound to specific, structured substates with characteristic contact patterns and distinct binding energies. In contrast, binding to the C-terminal region of α-synuclein was governed by a dynamic shuttling mechanism, where ligands sample similar interactions across a broad, low-barrier free-energy landscape, with moderate correlations between structural features (e.g., bend angle) and binding affinity.^36^ Those findings are consistent with our binding path framework. While we emphasize kinetic modeling using MSMs to describe the stochastic evolution of binding contacts, that work focused on clustering and thermodynamic characterization of conformational ensembles.^36^ Both studies converge on the conclusion that ligand binding to disordered proteins involves multiple, kinetically or structurally linked states, and both highlight the relevance of aromatic residues and surface accessibility in determining binding specificity. The results reported in that work further reinforce the notion that binding can be selective without being rigid, supporting the idea that recognition in disordered proteins relies on a continuum of favorable interactions rather than well-defined pockets,^36^ an insight foundational to the binding path concept.

Our framework is also related to the established use of MSMs to describe protein dynamics. Classical applications construct states from global conformational coordinates (distance or internal-coordinate clustering, principal-component projections, or related dimensionality reductions) and have proven powerful for folding and conformational change, where the slow modes correspond to large-scale structural rearrangements. Bowman and Pande showed that such conformational MSMs organize folding around a small number of kinetic hub states,^50^ Jain and Stock identified metastable states of folding peptides by clustering in a reduced internal-coordinate space,^51^ and Huang and co-workers applied the same conformational-state strategy to a disordered peptide, resolving a large number of weakly populated metastable conformations relevant to aggregation^52^. Our construction differs in three respects. First, the state variable is not directly a protein conformation but the ligand contact set (the n-plet of residues simultaneously engaging the small molecule), which captures the relevant slow mode present in these systems, namely, the redistribution of contacts as the ligand glides across a chain that remains conformationally heterogeneous within every binding state (Figure S4). Second, the same construction yields both thermodynamic and kinetic observables, including equilibrium populations, an MSM-derived K_D_, the gliding-versus-hopping decomposition (Figure 4), and the degree-centrality/affinity correspondence (Figure 6c). Third, because the model retains temporal ordering, a binding path is the time-ordered sequence of contact states the ligand visits rather than a static partition, and it is this ordering that motivates the design implications discussed below. The contact-state definition is therefore complementary to, rather than a relabeling of, conformational-MSM constructions, and to our knowledge, it has not previously been applied to small-molecule binding on disordered proteins.

We also note another recent study that demonstrated the utility of MSMs to elucidate the folding-upon-binding mechanisms of disordered proteins, specifically in the case of the measles virus NTAIL domain binding to its structured partner XD.^45^ Their MSM, constructed using a physically constrained VAMPNet with a multi-input neural network architecture, captures a network of kinetically distinct conformational intermediates characterized by progressive α-helix formation and native contact stabilization. In contrast, our MSM framework, designed to study small-molecule interactions with disordered proteins, does not resolve folding events but instead focuses on the stochastic rearrangement of residue contacts, termed binding paths, that describes diffusion-like ligand motion. While that MSM is suited to systems involving substantial structural reorganization upon binding, our MSM is optimized for ligands interacting with highly dynamic, unfolded ensembles where binding does not induce folding. Together, these two frameworks underscore the mechanistic diversity of ligand interactions with disordered proteins and highlight the importance of tailoring MSM constructions to the underlying biophysical context.

We anticipate that these findings will have implications for drug discovery targeting disordered proteins. By shifting the analysis from binding pockets to binding paths, we open the possibility of identifying targetable regions that may have been overlooked by using conventional methods. This change in perspective may ultimately contribute to the development of more effective therapeutic strategies for currently undruggable protein targets.

Taken together, the binding paths identify ranked druggable regions, mechanistic fingerprints that compare ligands by path diversity and flux, and kinetic bottlenecks that reveal contacts most likely to control the affinity and residence time.

## Conclusions

We introduced a stochastic framework for understanding and characterizing the interactions between small molecules and disordered proteins in terms of a disordered binding mechanism. By shifting from a static view of binding pockets to a dynamic view of binding paths, we offer a perspective that captures the transient and flexible natures of these systems. This approach offers insights into the design of small molecules with high binding affinity for disordered proteins, thus opening up promising directions for both fundamental research and the development of novel therapeutic strategies.

## Materials and Methods

### Analysis of Binding Pockets Using VAMPNet

Clustering binding pockets using VAMPNet involves an integration of machine learning and dynamic modeling.^46^ The input to this process consists of time-series data representing the distances between the center of mass of the ligand and each Cα atom of Aβ42 throughout a 28 μs MD simulation of the peptide in complex with the ligand G5. VAMPNet is used to extract slow collective variables (CVs) that capture the essential dynamical features of the system. These CVs are learned by training a neural network specifically optimized to model slow transitions between states. Once identified, the CVs are projected into a latent space, where transitions between configurations are approximated using a Markov process. This enables a kinetic description of the underlying conformational dynamics of the system. Clustering is then performed hierarchically in this latent space. The latent space is first divided into four coarse-grained clusters, which correspond to metastable states of the binding pocket. Each of these primary clusters is further subdivided into finer clusters to capture more granular features of the binding interactions (Figure 1). This hierarchical approach facilitates an efficient and dynamic classification of binding pocket configurations and provides insight into their structural and functional diversity. The VAMPNet model was trained in two stages: (i) the first stage used a lag time of 5 frames (50 ps), 4 states, a learning rate of 5 × 10^–3^, and 30 epochs, with a batch size of 10,000 (Figure S7). (ii) The second stage, which refined the model hierarchically, used a lag time of 10 frames (100 ps), the same number of states and learning rate, and 30 epochs, but with a reduced batch size of 500. To assess the reproducibility of the VAMPNet clustering, we performed five independent training runs and compared the resulting state assignments using adjusted mutual information scores (Figure S8).

### Calculation of the Handoff Matrix

Protein–ligand interactions were analyzed using MD simulations. Protein structures were processed with the MDAnalysis library^53^ to isolate the ligand and the relevant protein residues. To identify contacts, we measured the distances between all atoms of the ligand and protein in each simulation frame. A contact was defined as present when the distance between any two atoms (one from the ligand and one from a residue) was less than 3 Å (Figure 3). To characterize how the ligand moves between different binding regions on the protein, we divided the analysis into three distinct cases (Figure 3): Case A: handoff events in which residue *i* was in contact with the ligand at time *t* and residue *j* was in contact at time *t+*1, regardless of whether residue *i* maintained its contact. Case B: handoff events where residue *i* lost contact with the ligand and residue *j* gained contact at time *t+*1. Case C: events where residue *i* retained contact with the ligand, while residue *j* additionally gained contact at time *t+*1. These transitions were compiled into handoff matrices, which summarize the frequency of contact transitions between residue pairs. The matrices were visualized using heatmaps, providing insight into the dynamics of ligand movement and the identification of binding hotspots on the disordered protein.

### Clustering of Residues via Enumeration

States in the MSM are determined using a clustering-by-enumeration approach, which involves identifying residue groups (n-plets) that are in contact with the ligand at each time step based on spatial proximity. To carry out this analysis, we processed the protein–ligand interaction data by computing pairwise distances between ligand atoms and protein residues across all frames of an MD simulation. A contact was considered present if the distance between any pair of atoms fell below a defined cutoff. Two sources of distance information, one based on the center of mass of the ligand and the other on proximity to specific residues, were combined using weighted sums to enhance the resolution of contact detection. For each time frame, we then calculated the number of contacts and identified the most frequent n-plet type (i.e., a group of 2, 3, 4, or 5 residues in simultaneous contact with the ligand). These n-plets were considered discrete states, and a transition matrix was built by counting the frequency of transitions between them over time. To correct for enhanced sampling biases in the Aβ42 simulations, transition counts were weighted by the corresponding frame weights (see Supporting Information). To improve the quality of the model, duplicate states were filtered out, and an explicit unbound state was introduced at both the beginning and end of the trajectory to avoid creating artificially absorbing states. This enumeration-based approach provides a detailed and systematic way to define dynamic contact states, forming the basis for analyzing state transitions and quantifying binding affinities in disordered protein–ligand systems.

### Calculation of the Transition Probability Matrix from Molecular Dynamics Simulations

In this analysis, we describe an MSM that represents the transitions between different binding states, each defined as an n-plet of residues (e.g., quadruplets or quintuplets) simultaneously interacting with the ligand. Each binding state is assigned an index *i* = 1*,···,n*, and the probability of the system being in state *i* at time *t* is denoted as π_*i*_(t). These probabilities are assembled into a state probability vector π­(*t*) = [π_1_(*t*), ...,π_*n*_(*t*)]. The time evolution of this probability vector is governed by the master equation, which is a system of linear differential equations that describes how the population of each binding state changes over time due to stochastic transitions. We assume that the dynamics are Markovian (i.e., with no memory), time-independent, and linear, with constant transition rates. To estimate the transition rates from simulation data, we evaluate from the MD simulations an empirical population vector *p*(*t*), which approximates the population vector π­(*t*) in the MSM. We thus can obtain an MD-based estimate of the transition probability matrix T of the MSM by following the changes of *p*(*t*) upon a short time increment Δ*t*

2 p(t+Δt)≈p(t)T

### Graph Network Clustering

Based on the results of the Chapman–Kolmogorov test, which indicated that models using quintuplets provided the most stable MSM, we constructed a network graph G to visualize the connectivity between binding states. This graph was created using the NetworkX library, a Python library for creating, manipulating, and analyzing complex networks (graphs),^54^ providing data structures for nodes and edges as well as a wide range of built-in algorithms for tasks like path finding, clustering, and network statistics. In our graph, each node represents a quintuplet state, and each weighted edge represents the transition frequency between two states (Figure 6a). To prevent artifacts, self-loops (transitions from a state to itself) were removed from the graph. We applied Louvain clustering (with resolution = 3) to identify communities of closely connected states. Each node was colored according to its assigned community. The size of each node was scaled by its degree centrality, which was previously shown to correlate with the state’s binding affinity (Figure 6c). To enhance visual clarity, node positions were arranged using a spring layout, and edges were drawn with reduced opacity to avoid clutter. To quantify the importance of each community, we summed the equilibrium probabilities Peq of all nodes within that community. A sigmoid function was then applied to these summed probabilities to scale the visual representation of community importance (Figure S9). Finally, a supergraph was constructed to connect the communities, with individual subgraphs positioned relative to the supergraph layout (Figures 6b and 7), providing a high-level view of how groups of binding states are organized and interrelated in the MSM.

## Supplementary Material



## Data Availability

https://github.com/louetadelie1/MSM.git

## Notes

Supplementary methods, including molecular dynamics simulation protocols, sigmoid data transformation, binding affinity calculations, VAMPNet analysis, and trajectory reweighting; contact maps of the VAMPNet microstates; schematic of the Markov state model framework; Jaccard similarity analysis of transitions across 45 IDP–ligand systems; structural variability of the Louvain macrostates; biophysical determinants of binding-state stability; DSSP propensities of high-affinity states; VAMPNet convergence and reproducibility; sigmoid parameter selection; and table of the most frequent quintuplet-state transitions (PDF)
