# Supplementary material for: Binding without Docking: Small-Molecule Binding Paths on Disordered Proteins
Source: J Am Chem Soc. 2026 Sep 29;148(40):42969–80. doi: 10.1021/jacs.6c05891 (PMC13674065; doi:10.1021/jacs.6c05891)
Supplement: Supplementary file 1 [file ja6c05891_si_001.pdf]

SUPPLEMENTARY INFORMATION

Binding Without Docking: Small Molecule Binding Paths on Disordered Proteins

Adelie Louet[1,2,3], Gerhard Hummer[1,2], and Michele Vendruscolo[3,*]

[1]*Department of Theoretical Biophysics, Max Planck Institute of Biophysics, 60438 Frankfurt am Main, Germany*
[2]*Institute of Biophysics, Goethe University Frankfurt, 60438 Frankfurt am Main, Germany*
[3]*Centre for Misfolding Diseases, Yusuf Hamied Department of Chemistry, University of Cambridge, Cambridge CB2 1EW, UK*

Supplementary Methods

Aβ42 simulations

We analysed previously reported all-atom metadynamic metainference simulations[1-2] of free and bound Aβ42[3]. These simulations were out using GROMACS 2018.3[4] with PLUMED 2.6.0-dev[5], employing the CHARMM22* force field[6] and TIP3P water model[7], following a previously reported protocol[3]. Forty-eight initial conformations were generated and equilibrated at 278 K, first for 500 ps in the NVT ensemble using the Bussi thermostat[8], then for 500 ps in the NPT ensemble using Berendsen pressure coupling[3]. Production runs were conducted in the NPT ensemble with the Parrinello-Rahman barostat[3]. Enhanced sampling was achieved via parallel bias metadynamics using well-tempered and multiple-walker schemes[3]. Bias factors were set to 24 and 49 for unbound and bound simulations, respectively[3]. The unbound system used six collective variables (CVs) for conformational sampling, while the bound system included 14 additional CVs for protein-ligand contacts and four for ligand dihedrals. Simulations totalled 27.8 µs (unbound) and 28.2 µs (bound).

α-Synuclein simulations

To assess the robustness of our model across peptides of different lengths, we applied our approach to a previously reported trajectory of the α-synuclein C-terminus in complex with small molecules[9]. Using the published 60 µs atomistic molecular dynamics simulation performed with the a99SB-disp force field[10], and following the same analysis protocol described above, we identified 38, 37, 39, 68 residue clusters and reconstructed the binding paths of four representative small molecules (ligands 40, 50, 12, and fasudil) across α-synuclein.

Sigmoid function for data transformation

To determine the most suitable sigmoid transformation for visualizing the relative importance of different communities in our dataset, we first constructed an inverse mapping of state groupings, clustering related elements together. For each group, the equilibrium probabilities P_{eq} of its constituent states were summed to generate input values for the transformation. We applied a sigmoid function of the form:

$$S(x) = \frac{A}{1+\exp(-k(x-x_0))} \tag{S1}$$

where A is the amplitude (scaling factor), k controls the steepness of the curve, and x_0 is the midpoint of the transition. Several parameter sets were tested to evaluate their effect on the transformed values. These outputs were visualized using scatter plots to compare the behavior of each transformation function and assess their ability to reflect meaningful differences in community importance (**Figure S9**). We selected A = 1000, x_0 = 0.025, and k = 200 as a practical choice that improved visual contrast over the observed probability range. This parameter set provided the most effective scaling, clearly differentiating communities while preserving the overall distribution characteristics of the original equilibrium probabilities.

Binding affinity calculations
The binding affinity was estimated from molecular dynamics (MD) simulations using two approaches: a frame-based occupancy method and an MSM-based method. Both methods were applied without assuming predefined binding pocket locations.

Frame-based occupancy method. The dissociation constant K_D was estimated by comparing the number of frames where the ligand was unbound (p_u) versus bound (p_b), and scaling by the simulation box volume V

$$K_D = \left(\frac{p_u}{p_b}\right)\frac{1}{N_A \cdot V} \tag{S2}$$

where N_A is the Avogadro number, and p_u and p_b are the fractions of trajectory frames in which the ligand is unbound or in contact with at least one residue cluster, respectively.

MSM-based method. The dissociation constant was also derived from the equilibrium populations π^{eq} obtained from the long-time limit of the master equation defined by the MSM using the transition probability matrix calculated from the MD simulations (Eq. 2). The overall K_D was then obtained by comparing the equilibrium probability of the unbound state π_u^{eq} to the aggregate equilibrium probability of all bound states $\pi_b^{eq} = \sum \pi_i^{eq}$

$$K_D = \left(\frac{\pi_u^{eq}}{\pi_b^{eq}}\right)\frac{1}{N_A \cdot V} \tag{S3}$$

States with higher equilibrium populations correspond to lower K_D values and therefore stronger binding affinity.

VAMPNet analysis
The first VAMPNet[11] run was performed with a lag time of 5 steps, targeting a reduced model of 4 macrostates. The network was trained for 30 epochs using a learning rate of 5×10^{-3} and a batch size of 10,000. A second VAMPNet refinement was then conducted hierarchically using the output of the first model. This second run used a lag time of 10 steps with the same number of target states (4), learning rate (5×10^{-3}), and number of epochs (30), but employed a smaller batch size of 500 to better capture finer features in the state transitions. Cluster output from the second round of VAMPNet training (lag time = 10) after 5 independent runs (**Figure S8**). The plot shows the consistency of state assignments within this cluster across runs. Adjusted mutual information (AMI) scores were computed to quantify agreement between runs, indicating stable and reproducible clustering behavior.

Reweighting simulations

To reweight the biased trajectories, the time-ordered trajectories from each replica were first combined into a single continuous trajectory using the gmx trjcat tool. Parallel-bias metadynamics (PBMetaD)[12] was used to apply biases, with the corresponding HILLS files capturing the Gaussians deposited by each replica at every simulation step. Reweighting was then carried out on the full concatenated trajectory using plumed driver, resulting in a weight assigned to each frame. This method ensures alignment between the trajectory of each replica and its bias history, enabling accurate computation of unbiased weights.

IDP Simulations for Jaccard Similarity Analysis

To assess the generality of the gliding mechanism across structurally and chemically diverse IDP-ligand systems, we performed all-atom MD simulations of 12 intrinsically disordered proteins (Aβ42, α-CGRP, FUS, huntingtin, medin, p53 N- and C-terminal domains, PACAP-27, PACAP-38, prion protein, tau, and TDP-43) each in complex with small molecule ligands drawn from the ZINC database[13]. Simulations were carried out using GROMACS 2025.1 at 298 K, with 16 replicas per system for a total simulation time of approximately 2 μs per replica. Proteins were parametrized with the AMBER a99SB-disp force field[10] and the corresponding TIP4P-D water model. Ligand parameters were derived using the SAGE OpenFF 2.0.0 force field[14]. The same MSM-based Jaccard similarity analysis pipeline described above was applied uniformly across all 45 IDP-ligand trajectories.

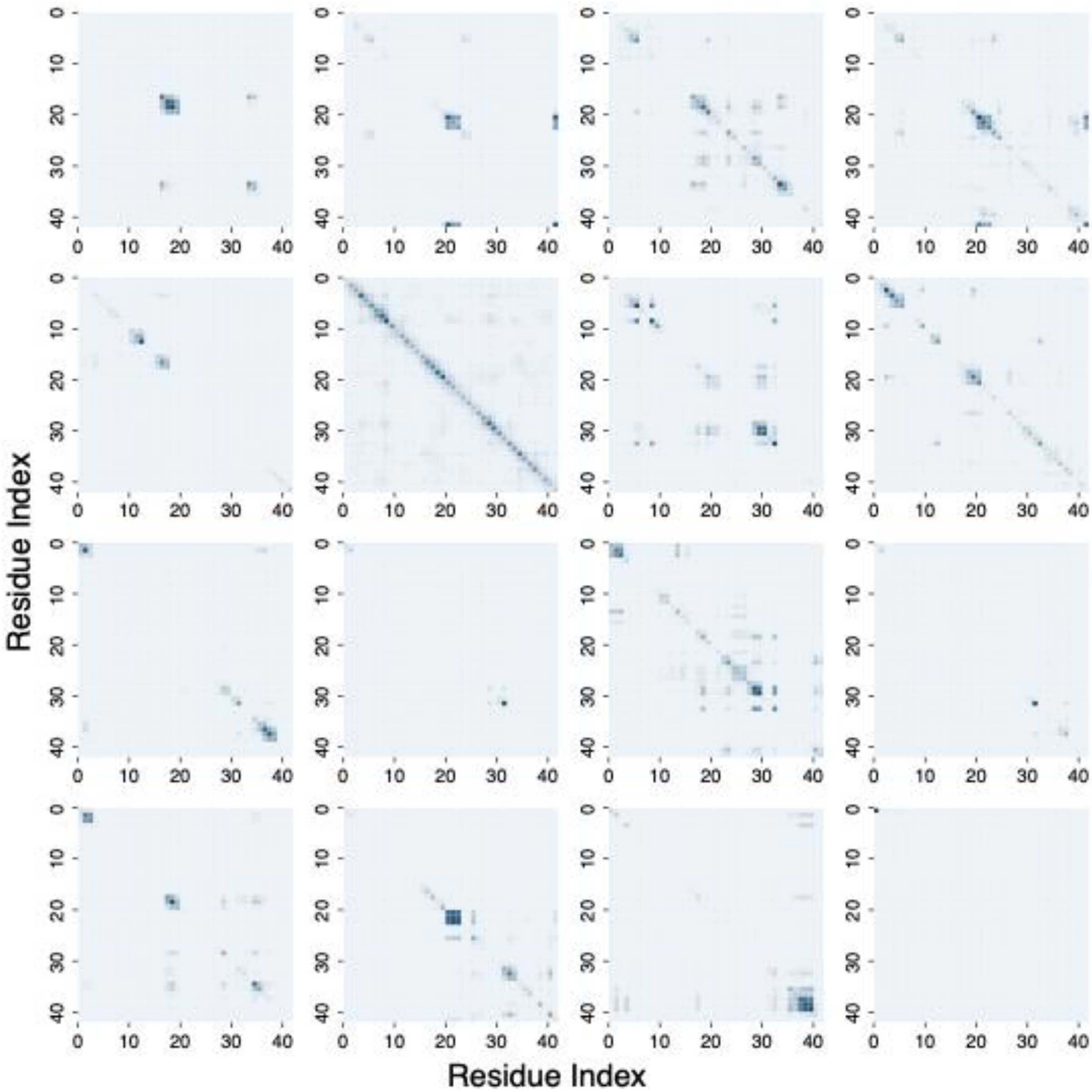

Figure S1. **Residue-residue contact maps for the 16 VAMPNet microstates of the Aβ42-G5 complex.** Each panel shows the mean pairwise contact frequency between Aβ42 residues for the conformations assigned to the corresponding cluster. The sparsely populated clusters exhibit sharply defined off-diagonal features, indicating specific residue-residue contacts characteristic of well-defined binding geometries. The dominant cluster (cluster 6 corresponds to cluster 2 Figure 1), which contains >90% of all frames (see main text), instead shows a diffuse pattern with no dominant contact pairs, consistent with a structurally heterogeneous ensemble that lacks a single preferred conformation. This complements **Figure 1**, which characterises the spatial relationship of a ligand with each cluster.

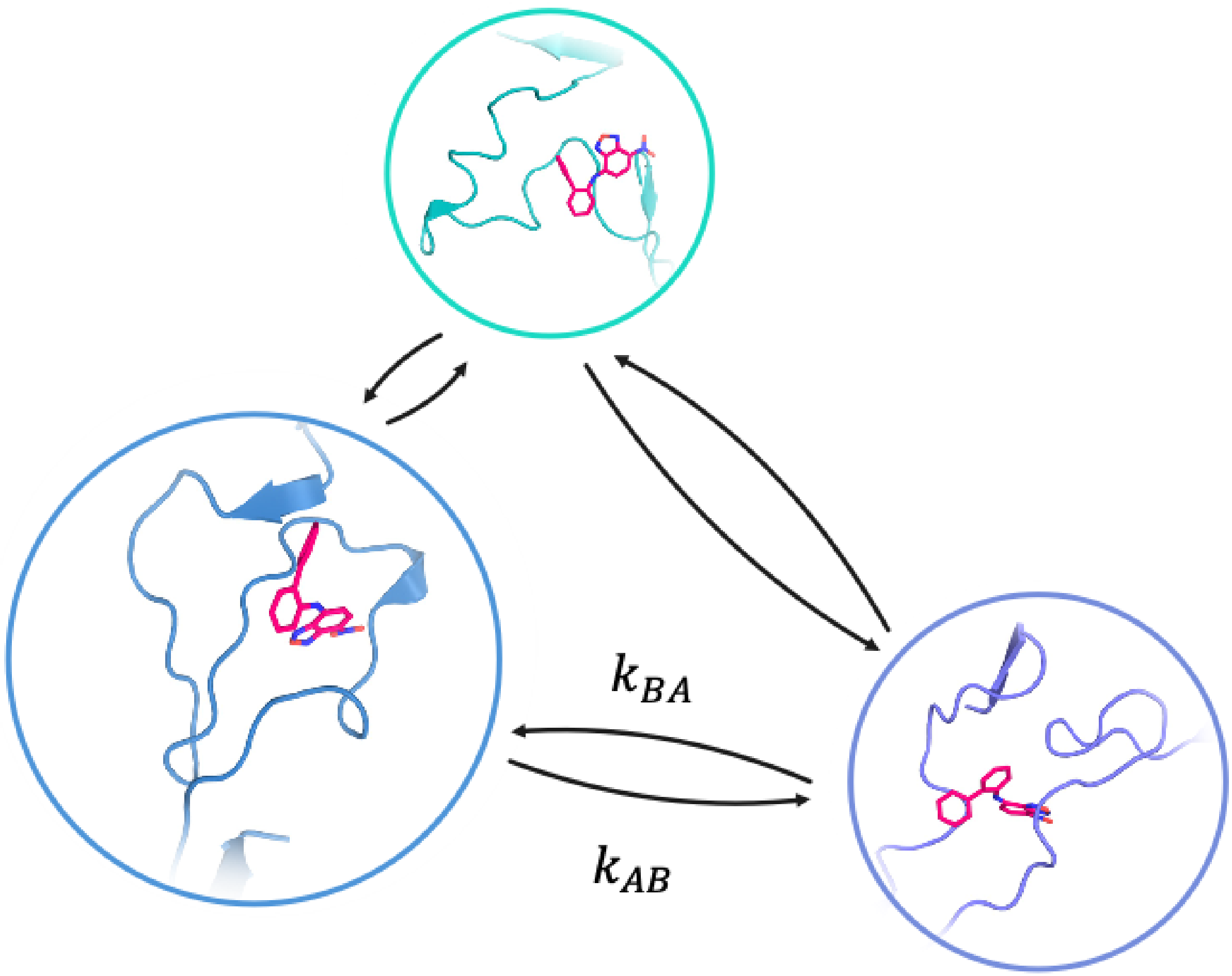

Figure S2. Conceptual illustration of the MSM framework for ligand binding to a disordered protein. Three representative snapshots from the Aβ42-G5 trajectory illustrate different MSM states, each defined by a distinct set of residues in contact with the ligand (red). Arrows indicate transitions between states, with the ligand moving to a different region in each. In the full model, the MSM comprises thousands of such states (13,034 quintuplet microstates for Aβ42) connected by a dense transition network (see **Figure 6a**); the three-state diagram shown here is a simplified schematic. The transition probability matrix is estimated from the MD trajectory and used to obtain the equilibrium population of each state (see Methods). Adapted from Lohr et al.[15].

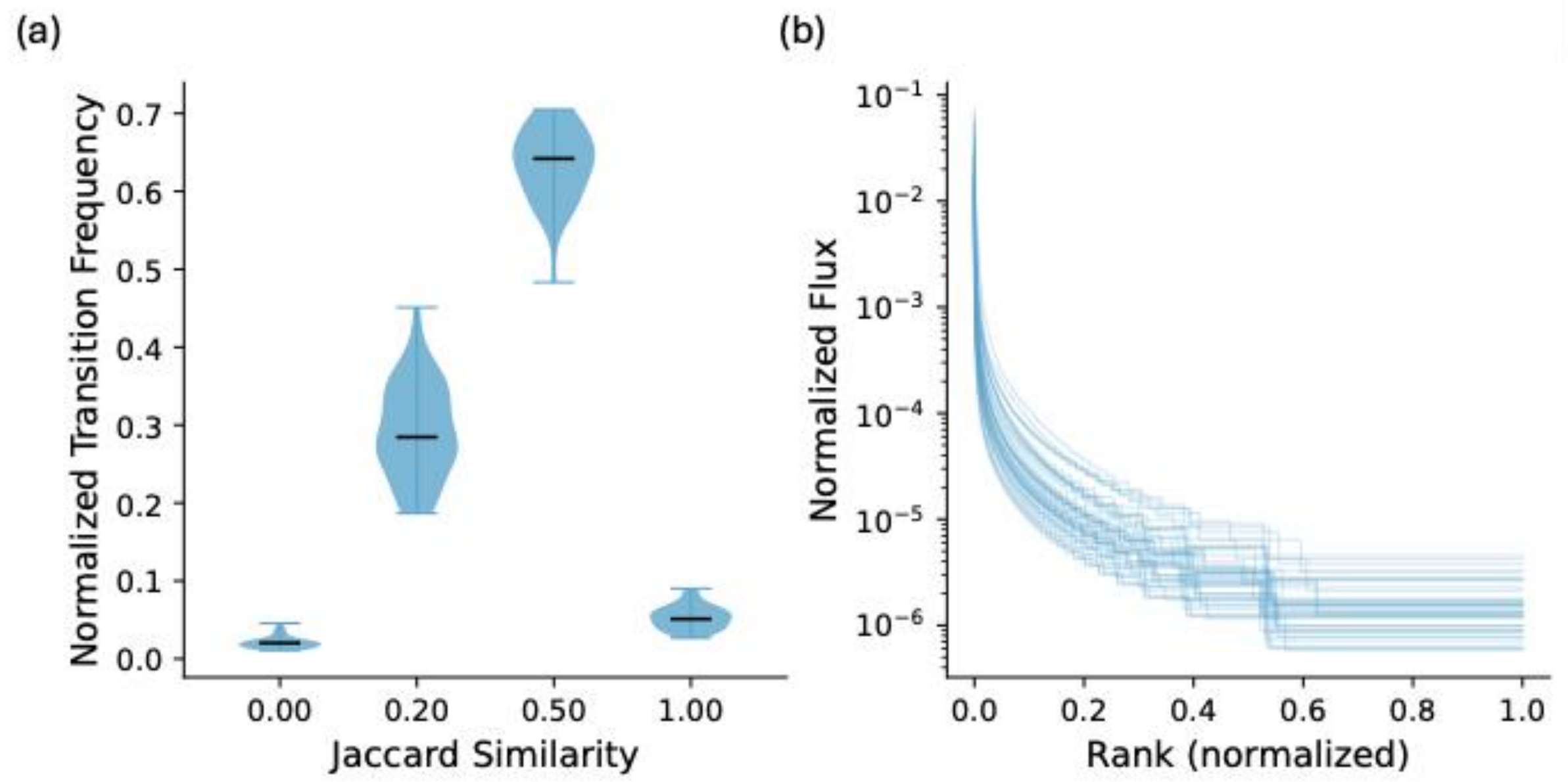

Figure S3. Hopping and gliding transitions across 45 IDP-ligand systems. (a) Distribution of Jaccard similarity scores for transitions observed across 45 IDP-ligand MD trajectories. Each violin shows the spread of normalised transition frequencies at a given Jaccard similarity value (0.0 = complete residue set exchange, hopping; 0.5 = partial overlap; 1.0 = identical contact set, gliding). The median is indicated by a black bar. **(b)** Rank-ordered normalised flux curves for all 45 systems. Each line represents one IDP-ligand trajectory, with transitions sorted from highest to lowest flux. The rapid decay across all systems indicates that a small number of high-flux transitions dominate the binding dynamics, while the extended low-flux tail reflects rare transitional events. Together, panels (a) and (b) demonstrate that gliding transitions (Jaccard = 1.0) consistently carry the dominant flux across chemically and structurally diverse IDP systems, establishing the generality of the binding-path mechanism beyond the Aβ42-G5 system characterised in the main text.

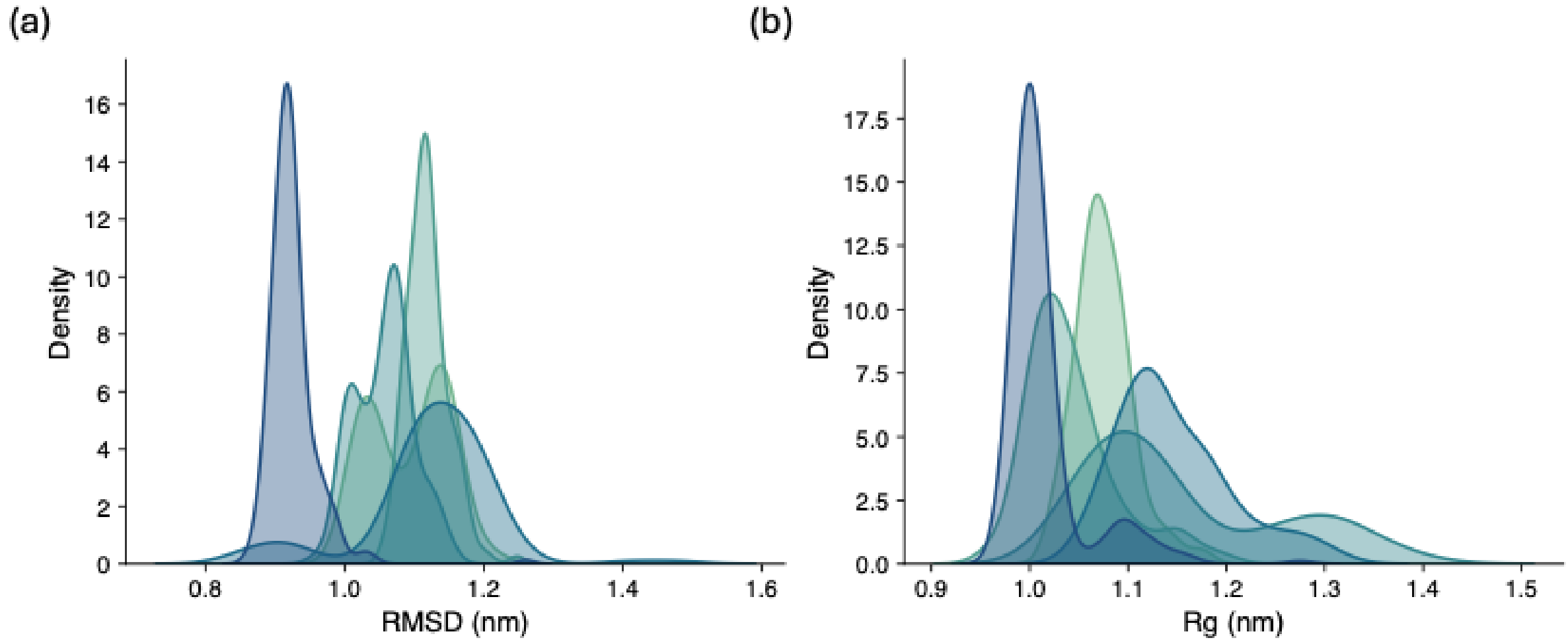

Figure S4. Structural variability within and between Louvain macrostate clusters. (a,b) Probability density distributions of the RMSD (a) and radius of gyration (Rg) (b) for five representative clusters. The distributions overlap extensively: the inter-cluster mean difference in RMSD (0.160 nm) is comparable to the intra-cluster standard deviation (0.130 nm), and similarly for Rg (0.110 vs 0.111 nm). This indicates that the MSM clusters, which are defined by ligand-residue contact patterns, do not correspond to structurally distinct protein conformations, as the protein remains conformationally heterogeneous within each binding state, consistent with the disordered nature of the complex.

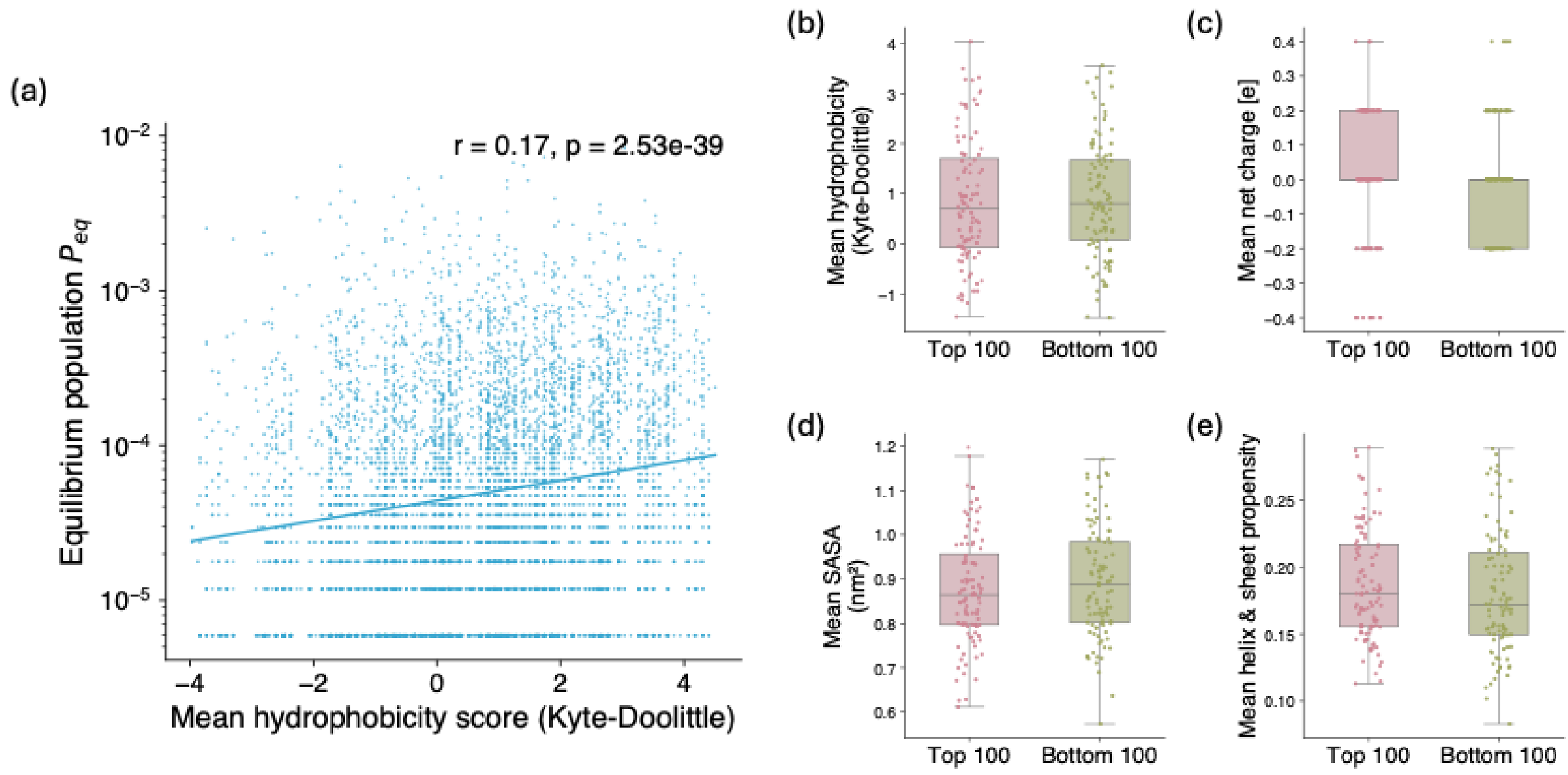

Figure S5. Biophysical features predict binding-state stability in the Aβ42-G5 system. (a) Equilibrium population (Peq) of each quintuplet microstate plotted against the mean Kyte-Doolittle hydrophobicity of its five contacted residues. Each point represents one of the 13,034 quintuplet microstates, with scatter points illustrating Peq on a logarithmic scale and the solid line showing the linear regression. The correlation is positive and statistically significant (Spearman $r = 0.17$, $p = 2.53 \times 10^{-39}$). The modest magnitude of r corresponds to hydrophobicity explaining approximately 3% of the variance in Peq, indicating that no single residue feature dominates binding and that high-Peq states emerge from the cooperative engagement of several properties. This is a quantitative signature consistent with the dynamic, multi-contact character of the binding mechanism. **(b-e)** Box plots comparing the top 100 and bottom 100 microstates ranked by Peq across four residue-level features computed as the mean over the five contacted residues of each quintuplet. **(b)** Mean Kyte-Doolittle hydrophobicity: the two groups have overlapping distributions (medians 0.7 vs. 0.8 - top vs. bottom), consistent with the small effect size identified in (a); the hydrophobicity contribution to binding is distributed across the state space rather than concentrated at the tails. **(c)** Mean net charge: the top 100 microstates show a broader distribution skewed slightly towards positive values, while the bottom 100 are tighter and skewed slightly negative. **(d)** Mean solvent-accessible surface area: high-affinity microstates have systematically lower SASA than low-affinity ones (medians 0.86 vs. 0.90 nm^2), indicating that strong binding is associated with arrangements in which the contacted residues are more buried than average. Mechanistically this indicates that the reduced SASA at the contact set is a signature of a transiently formed contact pocket rather than a featureless contact, and supports the interpretation of high-Peq states as locally compact arrangements that sequester the ligand against solvent. **(e)** Mean combined α-helical and β-sheet propensity (DSSP): high-affinity microstates show systematically elevated α-helical propensity compared with low-affinity ones (medians 0.18 vs. 0.17, respectively), a small but consistent shift consistent with the per-residue analysis in **Figure S6** and indicates that binding is associated with transient local ordering of the disordered chain. Together, both panels provide a quantitative biophysical basis for the state ranking in **Table S1**: high-affinity binding states tend to exhibit elevated hydrophobicity, reduced solvent exposure, and modest local ordering, with no single feature dominant and the affinity signal distributed across multiple chemical and geometric properties.

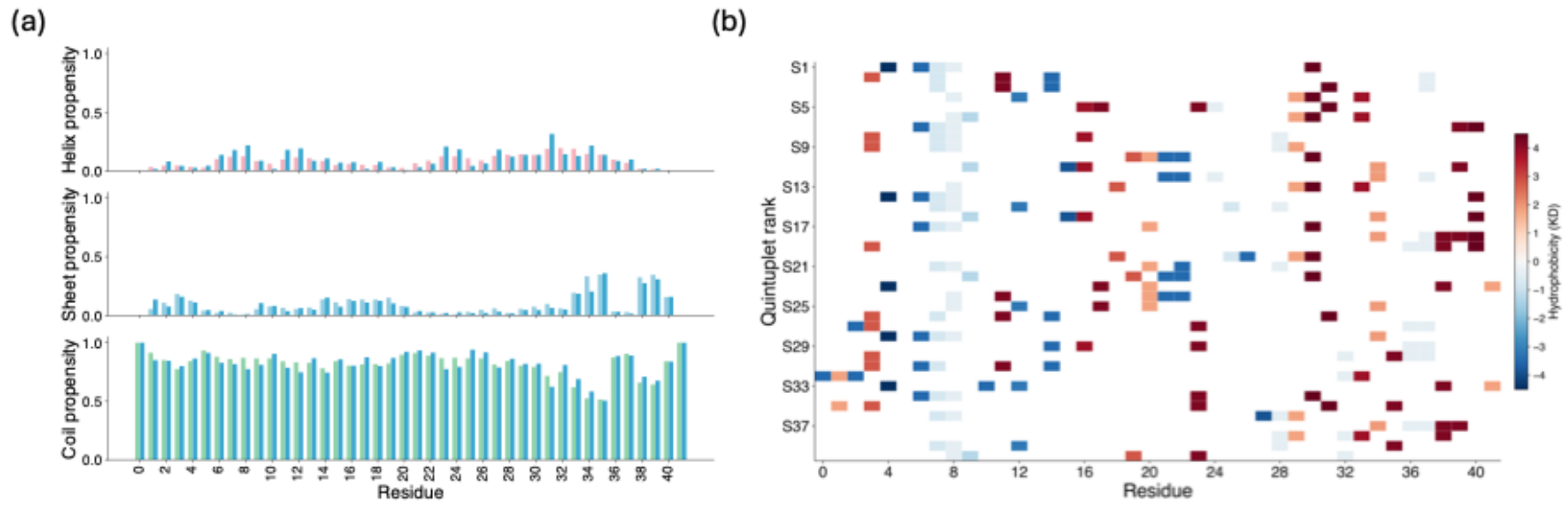

Figure S6. Residue-level and structural signature of high-affinity binding states in the Aβ42-G5 system. (a) Per-residue DSSP propensities for Aβ42. Each of the three subpanels reports one secondary-structure class (α-helix (top), β-sheet (middle) and coil (bottom)) and shows two bars per residue: the lighter bar gives the propensity averaged over all simulation frames, and the darker blue bar gives the propensity averaged over only those frames in which the residue is in contact with G5. Across the chain, the α-helical propensity is systematically elevated in the in-contact subset compared with the trajectory average, with the most pronounced increases at residues 18-23 and the C-terminal region (residues 32-38), and the β-sheet propensity is also enhanced in the in-contact subset at the C-terminal residues 35-40, where transient β-character is known to form, with a corresponding reduction in coil content. The systematic skew towards ordered secondary structure at ligand contacts indicates that G5 does not sample the disordered conformational ensemble uniformly but preferentially engages residues at moments when they adopt transient local order. This is a dynamic selectivity, since the same residues remain predominantly coil-like over the trajectory as a whole. Binding is therefore associated with transient secondary-structure formation on the disordered chain. **(b)** Residue composition of the 40 highest-Peq quintuplet microstates. Rows correspond to quintuplets ordered top to bottom by decreasing Peq, and columns correspond to Aβ42 residue positions 0-41. Cells are coloured by the Kyte-Doolittle hydrophobicity of the residue (red = hydrophobic, blue = hydrophilic) and left white when the residue is absent from the quintuplet. The visual signature is a marked concentration of hydrophobic contacts in the central hydrophobic cluster (residues 17-21) and the C-terminal region (residues 30-41), with these positions recurring in the majority of top-ranked quintuplets. Polar contacts at residues 5-8 and 22-28 appear in fewer than a third of the top quintuplets and are typically combined with hydrophobic contacts elsewhere in the same quintuplet. Together, panels (a) and (b) localise high-affinity binding on the Aβ42 sequence to specific hydrophobic clusters, and identify transient local ordering of the contacted residues as the dominant structural signature of binding events, connecting the residue-level identity of the high-affinity states (b) to the secondary-structure preferences that the ligand selects for at the moment of contact (a).

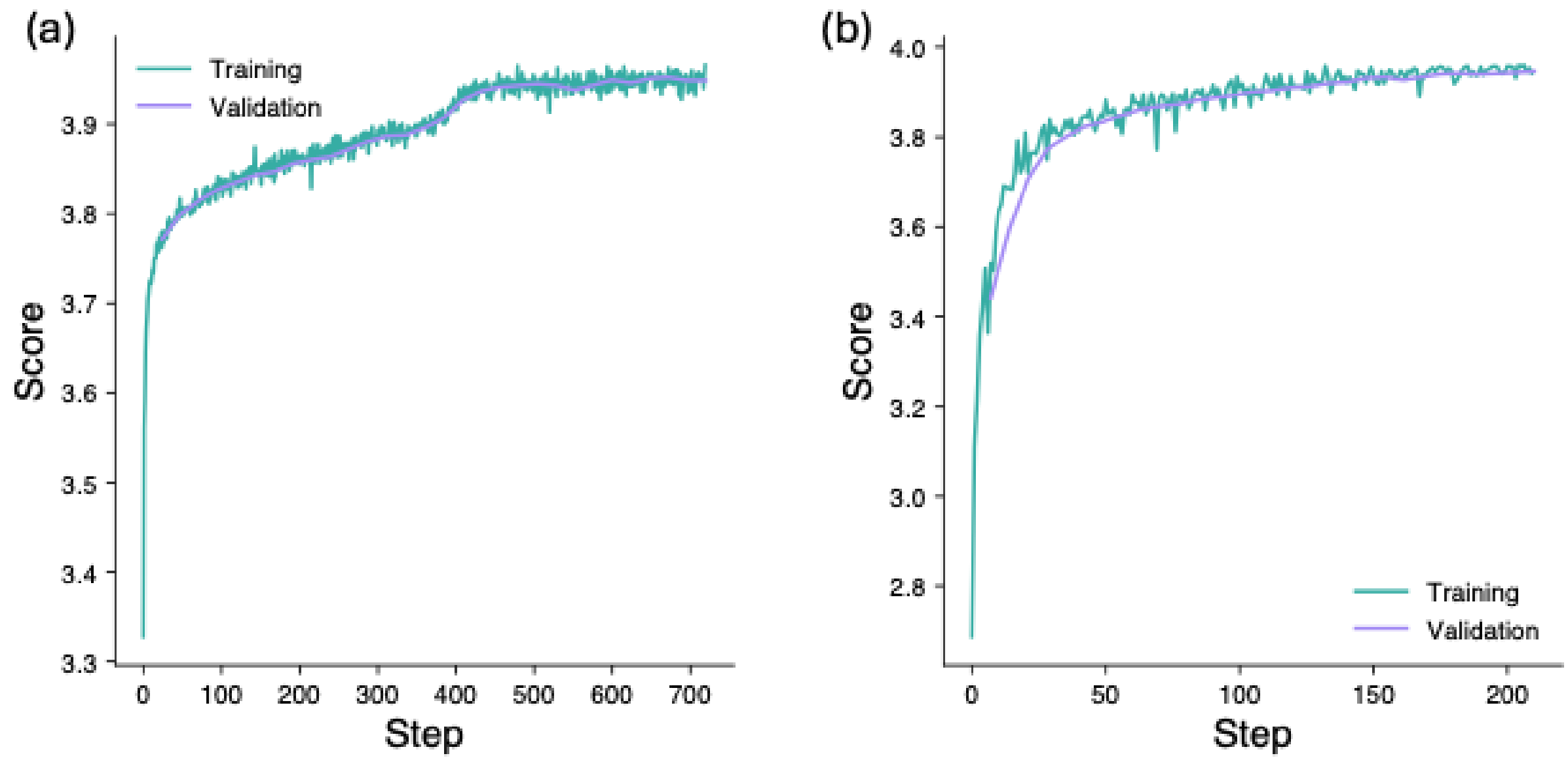

Figure S7. VAMPNet training convergence for one representative run. The VAMP-2 score, a variational metric quantifying the kinetic information captured by the model (higher is better), is plotted as a function of training step for both training (green) and validation (purple) datasets. **(a)** First hierarchical stage. **(b)** Second hierarchical stage. In both stages, training and validation scores converge within 200-300 steps and closely track each other thereafter, indicating stable optimisation with no evidence of overfitting. Curves from the remaining four independent runs are qualitatively similar.

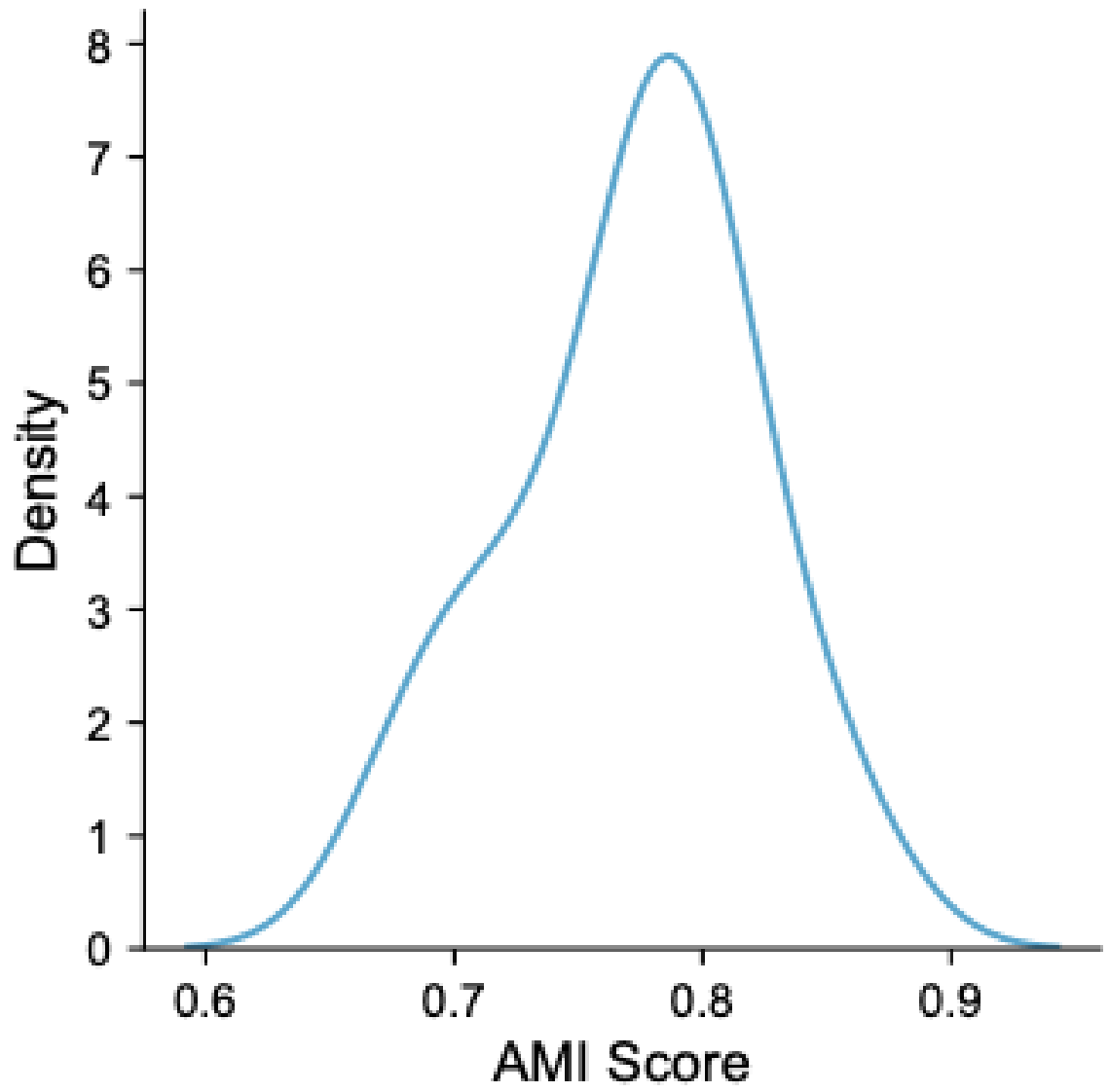

Figure S8. Reproducibility of VAMPNet state assignments across five independent training runs. The distribution shows the pairwise adjusted mutual information (AMI) scores between the clustering outcomes of all 5 pairs of runs, displayed as a kernel density estimate. AMI=1 indicates identical state assignments, and AMI=0 indicates chance-level agreement. The distribution peaks near 0.79 with a range of approximately0.6-1.0, indicating moderate but variable agreement between runs. This variability is consistent with the observation that VAMPNet struggles to resolve well-separated metastable states in this system (see **Figure 1** and main text).

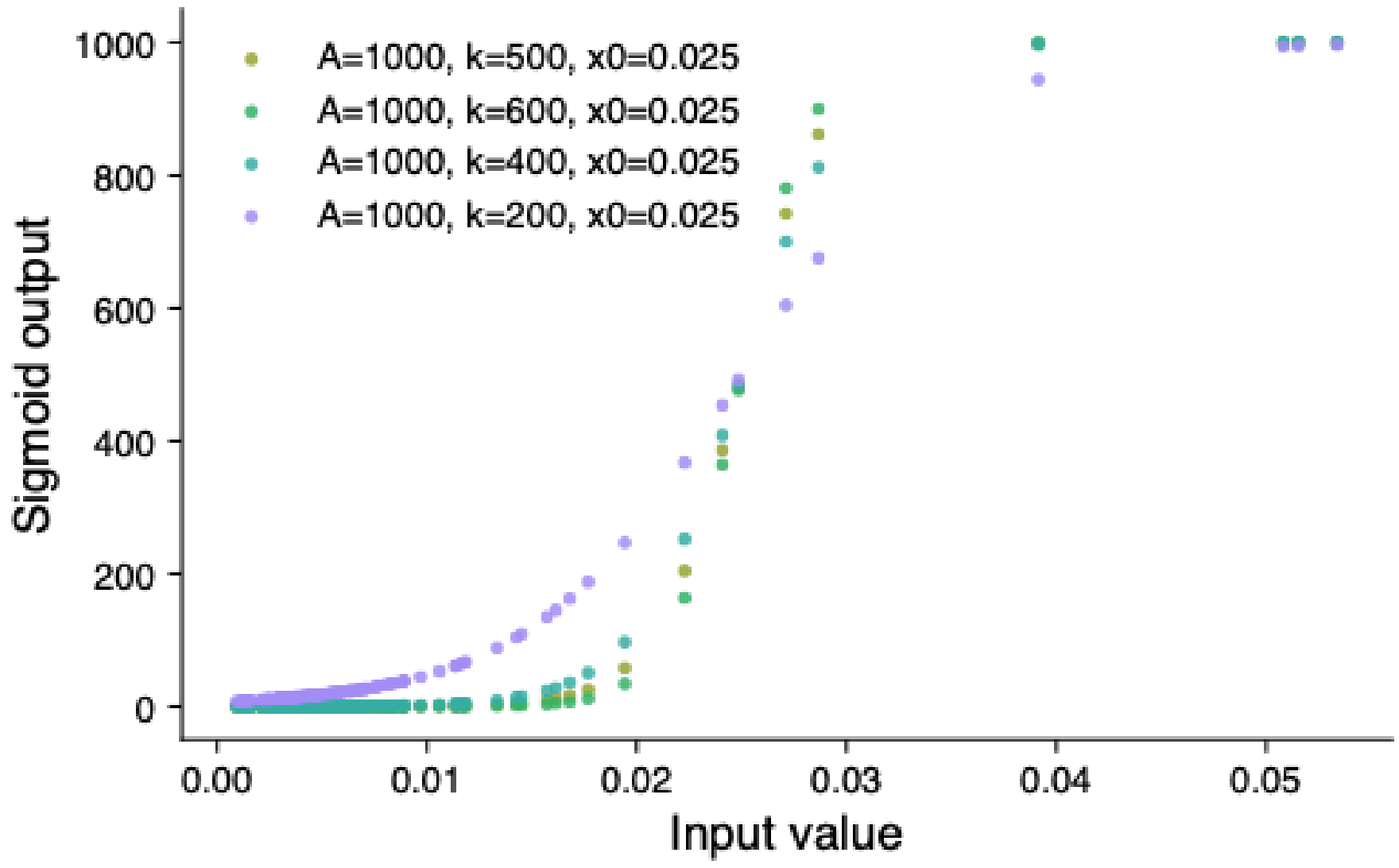

Figure S9. Selection of sigmoid steepness parameter for rescaling community weights. Each point represents one Louvain community; the x-axis is the summed equilibrium probability of the community over member states and the y-axis is the sigmoid-transformed value *S(x)* from Eq. S1, used to scale node sizes in **Figures 6b** and **7**. The amplitude (A = 1000) and midpoint ($x_0 = 0.025$) are held fixed; the four series show steepness values k = 200, 400, 500, and 600. k = 200 was selected as it provides the widest dynamic range across the observed probability distribution without saturating high-population communities.

Table S1. The 30 most frequent transitions between quintuplet states in the Aβ42-G5 MSM, ranked by normalised transition count. Each row reports one observed frame-to-frame transition between a "from" quintuplet and a "to" quintuplet, where a quintuplet is a set of five Aβ42 residues simultaneously in contact with G5; residue indices follow the Aβ42 sequence numbering used in **Figure S6**. T1-T10 are self-transitions, in which the ligand remains in the same quintuplet between consecutive frames (Jaccard similarity = 1.00); T11-T30 are inter-state transitions. For each transition the table lists: the Jaccard similarity between the from and to quintuplets (1.00 = identical state; 0.67 = four residues shared, i.e. a single-residue exchange; 0.43 = three residues shared); the equilibrium populations P_{eq} of the from and to states, taken from the stationary distribution of the MSM transition matrix and given in units of 10^{-3}; and the corresponding state-specific affinity constants $K_{ie} = \pi_u/\pi_i$ (Eq. 1), where π_u is the equilibrium population of the unbound state. K_{ie} is a dimensionless ratio. Because K_{ie} is inversely proportional to P_{eq}, states with higher equilibrium population have lower K_{ie} and therefore stronger binding. All inter-state transitions involve high residue overlap (Jaccard ≥ 0.43, the majority at 0.67), consistent with the gliding mechanism characterised in **Figure 4**. Several transitions form forward-reverse pairs, whose populations and affinity constants are exchanged between the from and to columns (e.g. T11/T13, T12/T14, T15/T16), reflecting near-equilibrium reversibility. These populations allow the transitions to be additionally ranked by binding-state stability and affinity.

Label	From quintuplet	To quintuplet	Jac. sim.	P_{eq}^{from} ($\times10^{-3}$)	P_{eq}^{to} ($\times10^{-3}$)	K_{ie}^{from}	K_{ie}^{to}
T1	(4, 6, 7, 8, 30)	(4, 6, 7, 8, 30)	1.00	17.20	17.20	1.21	1.21
T2	(3, 7, 11, 14, 37)	(3, 7, 11, 14, 37)	1.00	12.90	12.90	1.62	1.62
T3	(6, 7, 8, 39, 40)	(6, 7, 8, 39, 40)	1.00	6.88	6.88	3.03	3.03
T4	(8, 12, 29, 30, 33)	(8, 12, 29, 30, 33)	1.00	9.05	9.05	2.30	2.30
T5	(9, 15, 16, 34, 39)	(9, 15, 16, 34, 39)	1.00	6.09	6.09	3.42	3.42
T6	(7, 11, 14, 31, 37)	(7, 11, 14, 31, 37)	1.00	9.17	9.17	2.27	2.27
T7	(3, 7, 8, 16, 28)	(3, 7, 8, 16, 28)	1.00	6.29	6.29	3.31	3.31
T8	(8, 9, 29, 30, 33)	(8, 9, 29, 30, 33)	1.00	7.27	7.27	2.87	2.87
T9	(21, 22, 24, 34, 37)	(21, 22, 24, 34, 37)	1.00	5.69	5.69	3.66	3.66
T10	(6, 7, 8, 20, 30)	(6, 7, 8, 20, 30)	1.00	4.34	4.34	4.81	4.81
T11	(4, 6, 7, 8, 40)	(4, 6, 7, 8, 30)	0.67	5.27	17.20	3.95	1.21
T12	(3, 7, 11, 14, 37)	(7, 11, 14, 31, 37)	0.67	12.90	9.17	1.62	2.27
T13	(4, 6, 7, 8, 30)	(4, 6, 7, 8, 40)	0.67	17.20	5.27	1.21	3.95
T14	(7, 11, 14, 31, 37)	(3, 7, 11, 14, 37)	0.67	9.17	12.90	2.27	1.62
T15	(9, 19, 21, 22, 30)	(19, 20, 21, 22, 30)	0.67	3.94	6.15	5.29	3.39
T16	(19, 20, 21, 22, 30)	(9, 19, 21, 22, 30)	0.67	6.15	3.94	3.39	5.29
T17	(4, 6, 7, 8, 30)	(4, 6, 7, 8, 23)	0.67	17.20	1.98	1.21	10.53
T18	(8, 18, 29, 30, 33)	(8, 12, 29, 30, 33)	0.67	5.63	9.05	3.70	2.30
T19	(4, 6, 7, 8, 30)	(6, 7, 8, 23, 30)	0.67	17.20	2.65	1.21	7.87
T20	(4, 6, 7, 8, 23)	(4, 6, 7, 8, 30)	0.67	1.98	17.20	10.53	1.21
T21	(9, 19, 21, 22, 27)	(19, 20, 21, 22, 30)	0.43	0.58	6.15	35.71	3.39
T22	(7, 8, 12, 28, 35)	(7, 8, 10, 12, 25)	0.43	2.36	0.60	8.85	34.48
T23	(16, 17, 24, 31, 34)	(16, 17, 20, 23, 24)	0.43	1.44	1.71	14.49	12.20
T24	(16, 17, 20, 23, 24)	(16, 17, 24, 31, 34)	0.43	1.71	1.44	12.20	14.49
T25	(6, 9, 11, 30, 33)	(9, 11, 20, 21, 30)	0.43	1.13	0.29	18.52	71.43
T26	(19, 20, 21, 22, 30)	(9, 19, 21, 22, 27)	0.43	6.15	0.58	3.39	35.71
T27	(3, 5, 8, 32, 33)	(3, 8, 9, 29, 33)	0.43	0.38	1.23	55.56	16.95
T28	(8, 9, 12, 29, 33)	(8, 18, 29, 30, 33)	0.43	1.40	5.63	14.93	3.70
T29	(9, 12, 13, 18, 19)	(9, 12, 13, 16, 17)	0.43	0.75	0.25	27.78	83.33
T30	(3, 6, 7, 11, 14)	(7, 11, 14, 31, 37)	0.43	2.88	9.17	7.25	2.27

Supplementary References

1. Bonomi, M.; Camilloni, C.; Cavalli, A.; Vendruscolo, M., Metainference: A Bayesian inference method for heterogeneous systems. *Sci. Adv.* **2016,** *2* (1), e1501177.

2. Bonomi, M.; Camilloni, C.; Vendruscolo, M., Metadynamic metainference: Enhanced sampling of the metainference ensemble using metadynamics. *Sci. Rep.* **2016,** *6* (1), 31232.

3. Heller, G. T.; Aprile, F. A.; Michaels, T. C.; Limbocker, R.; Perni, M.; Ruggeri, F. S.; Mannini, B.; Löhr, T.; Bonomi, M.; Camilloni, C., Small-molecule sequestration of amyloid-β as a drug discovery strategy for Alzheimer's disease. *Sci. Adv.* **2020,** *6* (45), eabb5924.

4. Abraham, M. J.; Murtola, T.; Schulz, R.; Páll, S.; Smith, J. C.; Hess, B.; Lindahl, E., GROMACS: High performance molecular simulations through multi-level parallelism from laptops to supercomputers. *SoftwareX* **2015,** *1*, 19-25.

5. The PLUMED Consortium, Promoting transparency and reproducibility in enhanced molecular simulations. *Nat. Methods* **2019,** *16* (8), 670-673.

6. Piana, S.; Lindorff-Larsen, K.; Shaw, D. E., How robust are protein folding simulations with respect to force field parameterization? *Biophys. J.* **2011,** *100* (9), L47-L49.

7. Jorgensen, W. L.; Chandrasekhar, J.; Madura, J. D.; Impey, R. W.; Klein, M. L., Comparison of simple potential functions for simulating liquid water. *J. Chem. Phys.* **1983,** *79* (2), 926-935.

8. Bussi, G.; Donadio, D.; Parrinello, M., Canonical sampling through velocity rescaling. *J. Chem. Phys.* **2007,** *126* (1).

9. Robustelli, P.; Ibanez-de-Opakua, A.; Campbell-Bezat, C.; Giordanetto, F.; Becker, S.; Zweckstetter, M.; Pan, A. C.; Shaw, D. E., Molecular basis of small-molecule binding to α-synuclein. *J. Am. Chem. Soc.* **2022,** *144* (6), 2501-2510.

10. Robustelli, P.; Piana, S.; Shaw, D. E., Developing a molecular dynamics force field for both folded and disordered protein states. *Proc. Natl. Acad. Sci. USA* **2018,** *115* (21), E4758-E4766.

11. Mardt, A.; Pasquali, L.; Wu, H.; Noé, F., VAMPNets for deep learning of molecular kinetics. *Nat. Commun.* **2018,** *9* (1), 5.

12. Pfaendtner, J.; Bonomi, M., Efficient sampling of high-dimensional free-energy landscapes with parallel bias metadynamics. *J. Chem. Theory Comput.* **2015,** *11* (11), 5062-5067.

13. Irwin, J. J.; Tang, K. G.; Young, J.; Dandarchuluun, C.; Wong, B. R.; Khurelbaatar, M.; Moroz, Y. S.; Mayfield, J.; Sayle, R. A., ZINC20—a free ultralarge-scale chemical database for ligand discovery. *J. Chem. Inf. Model.* **2020,** *60* (12), 6065-6073.

14. Boothroyd, S.; Behara, P. K.; Madin, O. C.; Hahn, D. F.; Jang, H.; Gapsys, V.; Wagner, J. R.; Horton, J. T.; Dotson, D. L.; Thompson, M. W., Development and benchmarking of open force field 2.0. 0: the sage small molecule force field. *J. Chem. Theory Comput.* **2023,** *19* (11), 3251-3275.

15. Löhr, Thomas, et al. "A kinetic ensemble of the Alzheimer's Aβ peptide." Nature Computational Science 1.1 (2021): 71-78.
